# Coexpression of HHLA2 and PD-L1 on Tumor Cells Independently Predicts the Survival of Spinal Chordoma Patients

**DOI:** 10.3389/fimmu.2021.797407

**Published:** 2022-01-25

**Authors:** Chao Xia, Wei Huang, Yun-Liang Chen, Hai-Bin Fu, Ming Tang, Tao-Lan Zhang, Jing Li, Guo-Hua Lv, Yi-Guo Yan, Zhi-Hua Ouyang, Nvzhao Yao, Cheng Wang, Ming-Xiang Zou

**Affiliations:** ^1^ The First Affiliated Hospital, Health Management Center, Hengyang Medical School, University of South China, Hengyang, China; ^2^ Department of Spine Surgery, The First Affiliated Hospital, Hengyang Medical School, University of South China, Hengyang, China; ^3^ Shenzhen Audaque Data Technology Co., Ltd., Shenzhen, China; ^4^ Department of Spine Surgery, The Second Xiangya Hospital, Central South University, Changsha, China

**Keywords:** spinal chordoma, immune checkpoint molecules, quantitative immunofluorescence, HHLA2, PD-L1, tumor infiltrating lymphocytes

## Abstract

**Background:**

Immunotherapy only achieves efficacy in some cancer patients, and less is known about other immune checkpoint molecules in chordoma. Here, we aimed to determine the expression of PD-L1, HHLA2, B7H3, IDO-1 and Galectin-9 in spinal chordoma and evaluated their association with tumor infiltrating lymphocytes (TILs), clinicopathological characteristics and survival of patients.

**Methods:**

Using multiplexed quantitative immunofluorescence (QIF), we simultaneously measured the levels of five different immune checkpoint molecules and major TIL subsets in 92 human spinal chordoma samples.

**Results:**

Tumor HHLA2 and PD-L1 were positive in 80.0% and 86.0% of cases, respectively. However, B7H3, IDO-1 and Galectin-9 positivity on tumor cells were only seen in 21.0% of cases, despite all showing predominantly stromal expression. Coexpression of these QIF markers in the tumor compartment was scarcely detected except for PD-L1 and HHLA2, which was observed in 69.6% of cases. While tumoral HHLA2 and stromal B7H3 expressions were associated with an aggressive tumor phenotype, suppressive immune response (specifically including elevated PD-1^+^ TILs level and decreased CD8^+^ TIL density) and poor prognosis, stromal levels of PD-L1 and Galectin-9 predicted the opposite outcomes. Importantly, HHLA2 and PD-L1 coexpression on tumor cells independently predicted both worse local recurrence-free survival and overall survival.

**Conclusion:**

These data provide a better understanding of the immunosuppressive mechanism in chordoma and may be useful for the development of combination or novel immunotherapy approaches aiming to improve therapeutic efficacy and survival.

## Introduction

Chordoma is a rare subtype of bone sarcoma with an incidence rate of approximately 0.8 per million cases and is postulated to arise from residual embryonic notochordal tissue (1–3). Chordoma is regarded as a low-grade neoplasm with a strong tendency toward local recurrence (4). Histologically, chordoma typically consists of physaliphorous cells that are arranged in cords or sheets within the abundant myxoid stroma (5). Chordoma is divided into four pathology subtypes: conventional, chondroid, dedifferentiated and poorly differentiated (6). Due to its unresponsiveness to conventional chemotherapy, the standard therapy for chordomas is surgery followed by adjuvant radiation therapy (7–9). However, total surgical resection of these tumors remains challenging because of the proximity to vital neurovascular structures. Therefore, the recurrence risk of chordoma is high after surgery, and 40-50% of patients may experience metastasis, which causes great pain to patients (10–13). Thus, the development of novel efficient therapy is urgently needed to improve patient survival.

With the increasing understanding of the immune microenvironment in tumor pathogenesis, immunotherapy has been widely applied in cancer patients. For example, the use of PD-1/PD-L1 or CTLA-4 inhibitors has displayed good efficacy in a variety of cancers (14–17). In chordoma, studies have demonstrated frequent immune cell infiltration in the tumor microenvironment and their significant association with patient characteristics and prognosis (18). Although these data suggest immunotherapy as a promising therapeutic strategy for chordoma, the results are still controversial, and data regarding the efficacy of immune checkpoint blockades in this disease are limited (19–22). Moreover, previous studies demonstrated that only 13-45% of solid cancer patients had a good response to immunotherapy, indicating alternative immunosuppressive checkpoints or pathways disrupting immunosurveillance (23, 24). Therefore, it is important to search for other novel immunotherapy targets.

Recently, researchers identified several other immune checkpoint molecules that are aberrantly expressed in cancers and can hamper antitumor immunity to promote tumor progression *via* various mechanisms (25–28), such as human endogenous retrovirus-H long terminal repeat-associating protein 2 (HHLA2), B7H3, indoleamine 2,3-dioxygenase (IDO-1) and galectin-9. These findings suggest that other immune checkpoint molecules represent additional immunosuppressive mechanisms within the tumor microenvironment, which may have implications for immunotherapy combinations or the development of novel immunotherapy strategies in the future. However, there is currently a lack of data evaluating the expression of these markers in chordoma. In this study, we sought to objectively measure the expression of PD-L1, HHLA2, B7H3, IDO-1 and Galectin-9 in spinal chordoma tissues by multiplexed quantitative immunofluorescence (QIF). We also attempted to analyze these data with clinicopathological features, the immune microenvironment and the survival of patients.

## Methods

### Patient and Tissue Samples

In this study, a total of 92 spinal chordoma patients who received surgical treatment at our hospitals were included. Seventy-seven (between June 2002 and December 2018) of these patients were previously described in our studies (29, 30) and 15 (between December 2009 and December 2016) were newly added from another institute. Patients with immune-related diseases or other tumors (specifically including 5 with rheumatoid arthritis, 1 with scleroderma, 4 with ankylosing spondylitis, 2 with prostate cancer and 1 with rectal cancer) on admission and those who had previously received tumor-specific therapy (specifically including radiotherapy, chemotherapy or immunotherapy) were excluded, considering that these conditions may affect the immune profile within the chordoma microenvironment. For example, trabectedin, a chemotherapeutic drug, plays an antitumor role in several types of soft tissue tumor, possibly by affecting the tumor microenvironment (31). All patients underwent surgery, and 20 recurrent patients had undergone preceding surgery on admission. Although prior surgery may cause inflammation to impact the immune phenotype and function, all recurrent patients were admitted to our institute more than half a year after their initial surgery. Therefore, we consider that inclusion of the recurrent patients in our study was appropriate and that their prior surgery could not have a significant influence on the results. Another reason for including recurrent patients is the fact that chordoma tends to relapse after surgery, and enrolling these patients is more representative of the chordoma population in the real world (29). Patients were categorized as Enneking appropriate (EA) when the final pathology margin matched the surgical margin as recommended by the Enneking classification (32); otherwise, they were categorized as Enneking inappropriate (EI). Other clinicopathologic characteristics were recorded and evaluated as we previously described (30). The tumor Ki-67 index and densities of microenvironmental tumor-infiltrating lymphocytes (TILs) (including PD-1^+^, CD3^+^, CD8^+^, CD20^+^ and Foxp3^+^ TILs) in 77 patients were directly retrieved from our prior data (29, 33). The Ki-67 and TIL data of 15 newly added patients were obtained by immunohistochemistry and QIF methods, respectively, in which the same staining procedures, antibodies (**Supplemental Table 1**) and dilution ratios were used to allow for comparability.

Ninety-two tissue specimens were retrieved from the Department of Pathology, and formalin-fixed paraffin-embedded (FFPE) blocks were sectioned into 4-μm thick tissue sections for subsequent assays. Two experienced pathologists were assigned to confirm the diagnosis based on the histological findings in H&E-stained tissue sections, according to the criteria described in a previous study (3). The study was approved by our hospital ethical committee, and informed consent was obtained from all patients.

### Follow-Up

Patients were followed-up by regular clinical and radiographical examinations until September 2020. Follow-up was arranged at three-month intervals in the first two years after surgery, then every six months for three years postoperatively, and annually thereafter (33). Patient outcomes were measured as local relapse-free survival (LRFS) and overall survival (OS). Observations were censored when a patient was tumor-free (LRFS analysis) or alive (OS analysis).

### Multiplexed Quantitative Immunofluorescence

**Figure 1:**
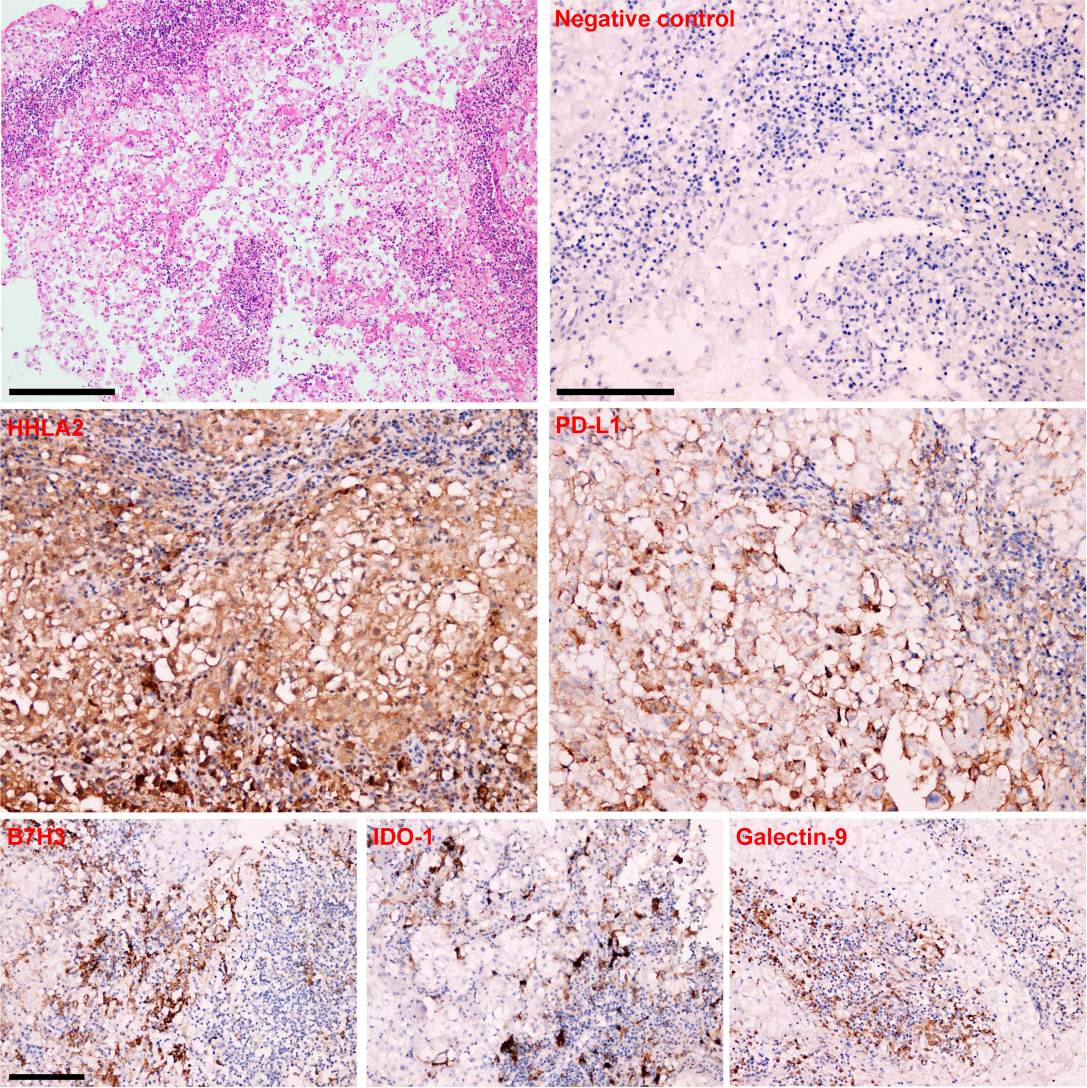
HE staining and Immunohistochemistry analysis in human spinal chordoma tissues. Representative immunohistochemistry images showing the negative control and positive expression of HHLA2, B7H3, IDO-1, PD-L1 and Galectin-9 in a chordoma sample. Scale bar = 100 μm.

QIF was performed using the Opal 7-color Manual IHC Kit (PerkinElmer, Waltham, Massachusetts) as we previously documented (29, 34). For each tumor tissue, we measured the levels of PD-L1, B7H3, HHLA2, IDO-1 and Galectin-9 using a sequential staining protocol with simultaneous detection of cytokeratin 19 and 4′,6-diamidino-2-phenylindole (DAPI; PerkinElmer), as described previously (34). To ensure the reliability of our QIF data, we performed immuno-histochemistry staining before the QIF assay to test the sensitivity and specificity of the antibodies in chordoma tissues (**Figure 1**). Briefly, the FFPE sections were deparaffinized followed by antigen retrieval in a pressure cooker with Tris-ethylenediaminetetraacetic acid buffer (pH 9.0) for 10 minutes. The tissue sections were then incubated with the primary antibodies at 4°C overnight, followed by antigen blocking with 3% H_2_O_2_ for 15 minutes and 10% goat serum for 30 minutes at room temperature. After this, horseradish peroxidase (HRP)-conjugated secondary antibodies were incubated at room temperature for 1 hour followed by tyramide-based HRP activation at 37°C for 20 minutes. Residual HRP activation was eliminated by 1 mM benzoic hydrazide with 0.15% H_2_O_2_. Goat anti-mouse HRP and Opal 690 were applied to reveal cytokeratin 19. Similarly, goat anti-rabbit HRP and Opal 520, Opal 540, Opal 570, Opal 620 or Opal 650 conjugates were used to detect B7H3, PD-L1, IDO-1, HHLA2 and Galectin-9, respectively. Finally, the slides were sealed with coverslips using ProLongGold Antifade reagent with DAPI and allowed to dry overnight.

QIF of PD-1^+^, CD3^+^, CD8^+^, CD20^+^ and Foxp3^+^ TIL subsets in 15 newly added tumor samples was detailed in a published work by our group (29). Briefly, each individual tissue section was stained with PD-1, CD3, CD8, CD20, Foxp3 and cytokeratin 19 using the standard protocol, with simultaneous quantitative measurement in both the tumor and stromal compartments using methods described previously (29). The antibody information is listed in **Supplemental Table 1**.

### Fluorescence Signal Quantification and Case Stratification

Images were analyzed by inForm software (version 2.1.1, PerkinElmer) using a previously described method (29). Briefly, images of the whole tumor section were captured using a Vectra system (version 2.0.8, PerkinElmer) scanner with a 4x objective under the same exposure time, laser power and bit depth to ensure comparability. After this, 10 areas of interest were selected for evaluation from images of each tumor sample. DAPI was used to identify cell nuclei. The tumor compartment was revealed as the regions with cytokeratin 19 positivity. The stromal compartment excluded the tumor mask from the DAPI compartment. The QIF scores of PD-L1, B7H3, HHLA2, IDO-1 and Galectin-9 (all in tumoral and stromal compartments) were calculated by dividing the target marker pixel intensities by the area of the corresponding mask (recorded as integrated optical density [IOD]/10^6^ pixels), similar to previous reports (27, 29). Marker was recorded as positive when its QIF score was above the signal detection threshold (specifically 3.435647 for PD-L1, 4.935647 for B7H3, 6.475462578 for HHLA2, 11.81259611 for IDO-1, and 6.114850256 for Galectin-9). We determined the signal detection threshold based on the overall expression pattern of the immune markers on QIF images of the whole tumor section and visual inspection (we checked visually for the staining pattern in each case) to exclude possible nonspecific staining and background signals, similar to a previous report (27). Of note, the QIF values below this detection threshold were not always zero (most commonly larger than 0), and we used these data in subsequent quantitative statistical analysis to ensure the accuracy of our results. Subsequently, the average from all areas of interest for each tumor was obtained for final analysis. All images were visually reviewed, and those with staining artifacts or less than 3% tumor tissue were excluded from the analysis.

### Statistical Analyses

Continuous data are summarized as the mean ± standard deviation and were analyzed by Student’s t test or One-Way ANOVA test. Categorical data are expressed as the frequency or composition ratio and were analyzed by the chi-square test or Wilcoxon’s rank sum test, when appropriate. Pearson’s correlation test was used to assess the relationship between continuous variables. Cutoff Finder Web Application (http://molpath.charite.de/cutoff) was used to obtain the threshold value (**Supplemental Figure 1**) for QIF data (including B7H3, HHLA2, IDO-1 and Galectin-9) in survival analysis with OS as the outcome parameter (29). In detail, the threshold value was determined as the point with the minimum *P* value from the log-rank test, which was subsequently corrected (35). QIF data of PD-L1 were separated into two groups as previously suggested (34). The Kaplan–Meier method was applied to display the LRFS and OS curves, and differences in survival probabilities among subgroups were analyzed by the log-rank test. Multivariate Cox proportional hazard models were used to identify independent factors associated with LRFS and OS after controlling for covariates that were significant in univariate analysis and previously reported significant predictors (29). Statistical analyses were performed by R version 3.5.1 (R Foundation for Statistical Computing, Vienna, Austria). All tests were two-sided, and a *P* value ≤ 0.05 was considered statistically significant.

## Results

### Patient Characteristics

**Figure 2:**
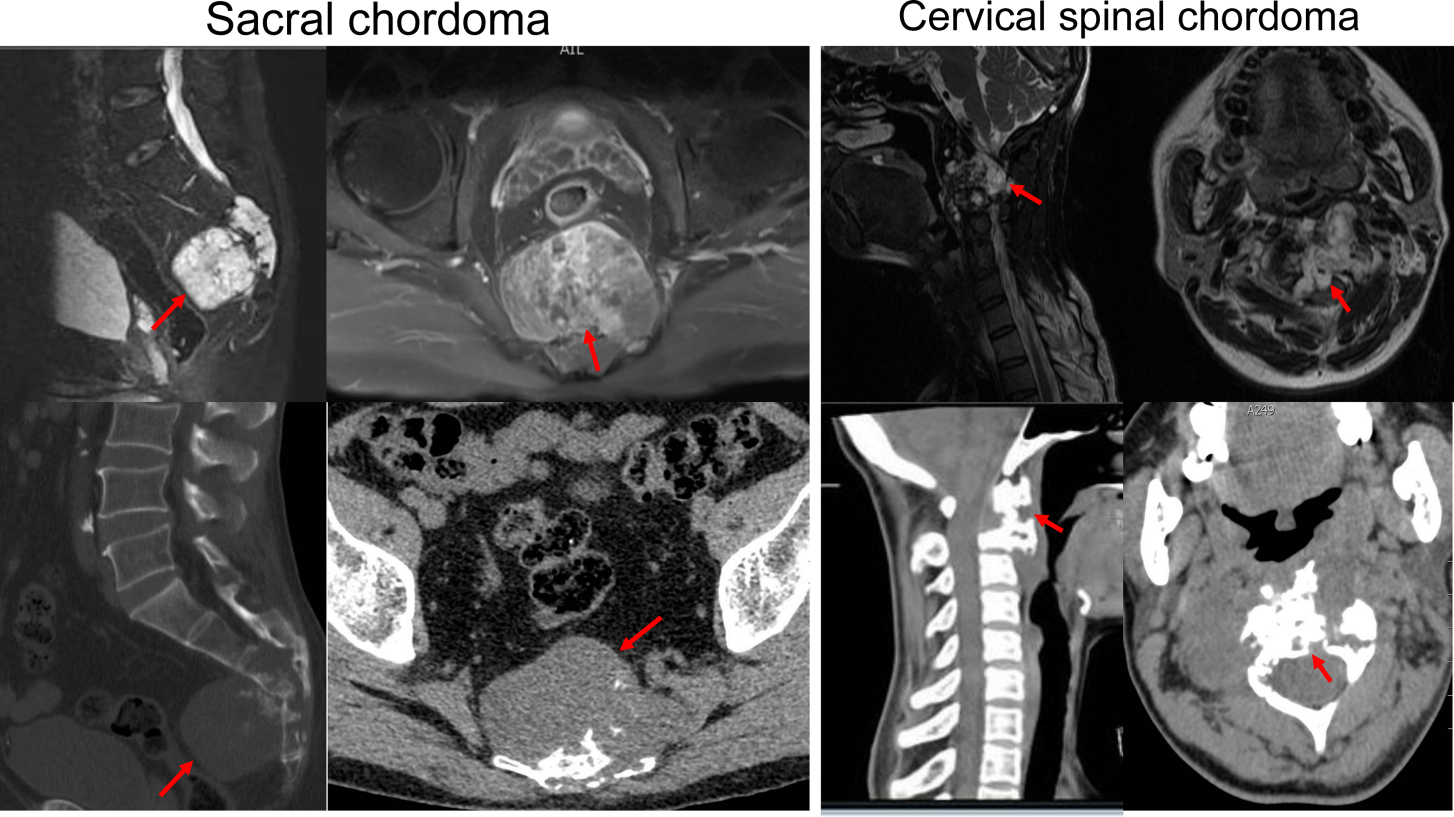
Representative images of MRI and CT scan of sacral and cervical chordoma. The red arrows indicate the location of chordoma.

A total of 92 patients were included. The clinicopathological characteristics of the patients are detailed in **Supplemental Table 2**. In brief, the cohort had 25 females and 67 males. Among them, 20 patients had recurrent chordomas, and 72 patients had primary diseases. A total of 34 patients underwent EI resection, and 58 underwent EA resection. There were 72 sacral chordomas and 20 chordomas located at the mobile spine (**Figure 2**). All chordoma cases were confirmed as conventional pathology type, and no dedifferentiated or poorly differentiated cases were included. Thirty-five chordomas displayed a lobular growth pattern, and 57 did not. Tumor grade was low in 31 cases and high in 61 cases as assessed by structural and nuclear atypia (32). The average follow-up time was 40.98 ± 35.52 months, and no patients were lost to follow-up. The median LRFS and OS were 19 months and 27.5 months, respectively.

### Expression and Coexpression of QIF Markers in Spinal Chordoma Tissues

**Figure 3:**
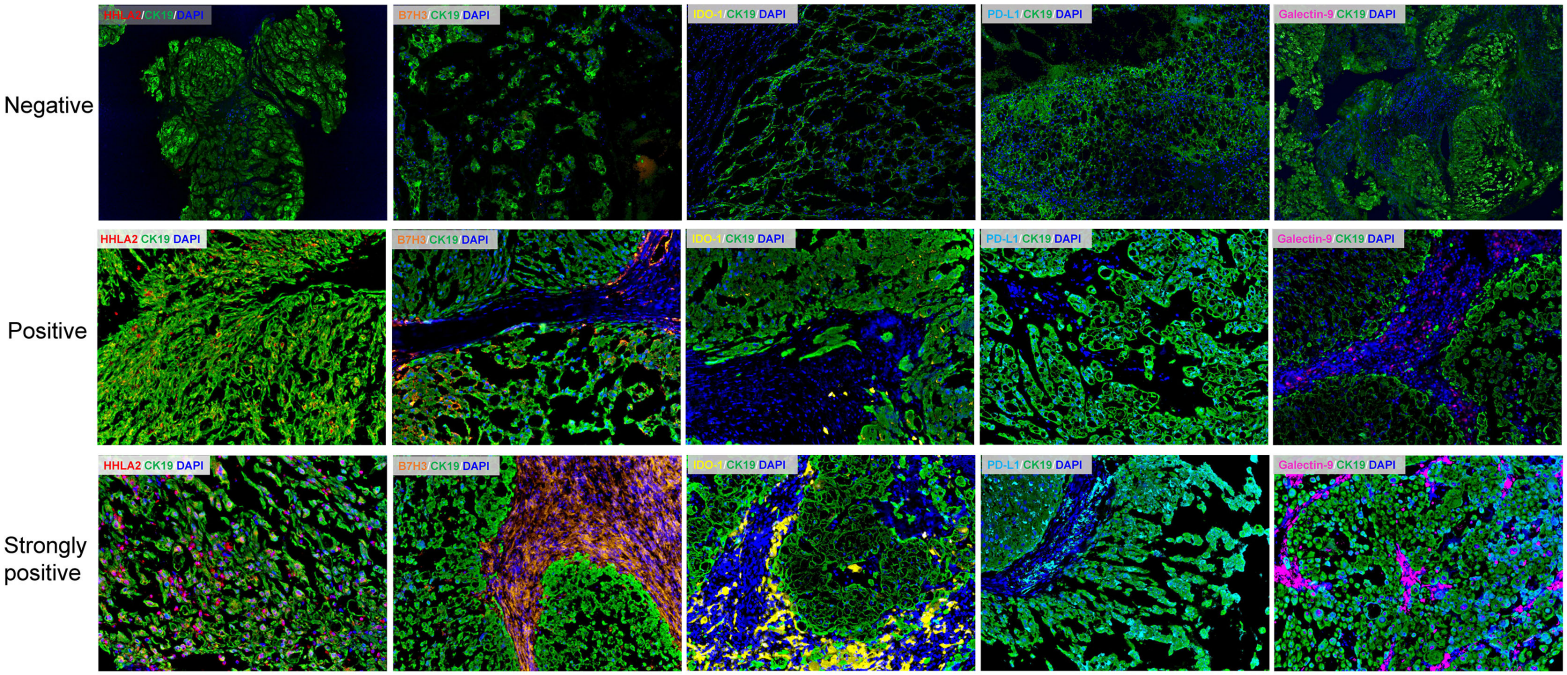
Representative immunofluorescence images showing negative, positive and strongly positive expression of HHLA2, B7H3, IDO-1, PD-L1 and Galectin-9 within the tumor and stromal subregions of chordoma tissues. Scale bar = 100 μm. Nuclei were stained with DAPI.

**Figure 4:**
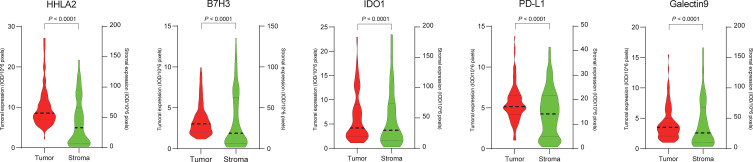
Comparison of the expression levels of HHLA2, B7H3, IDO-1, PD-L1 and Galectin-9 between the tumoral and stromal compartments of chordoma tissues.

**Figure 5:**
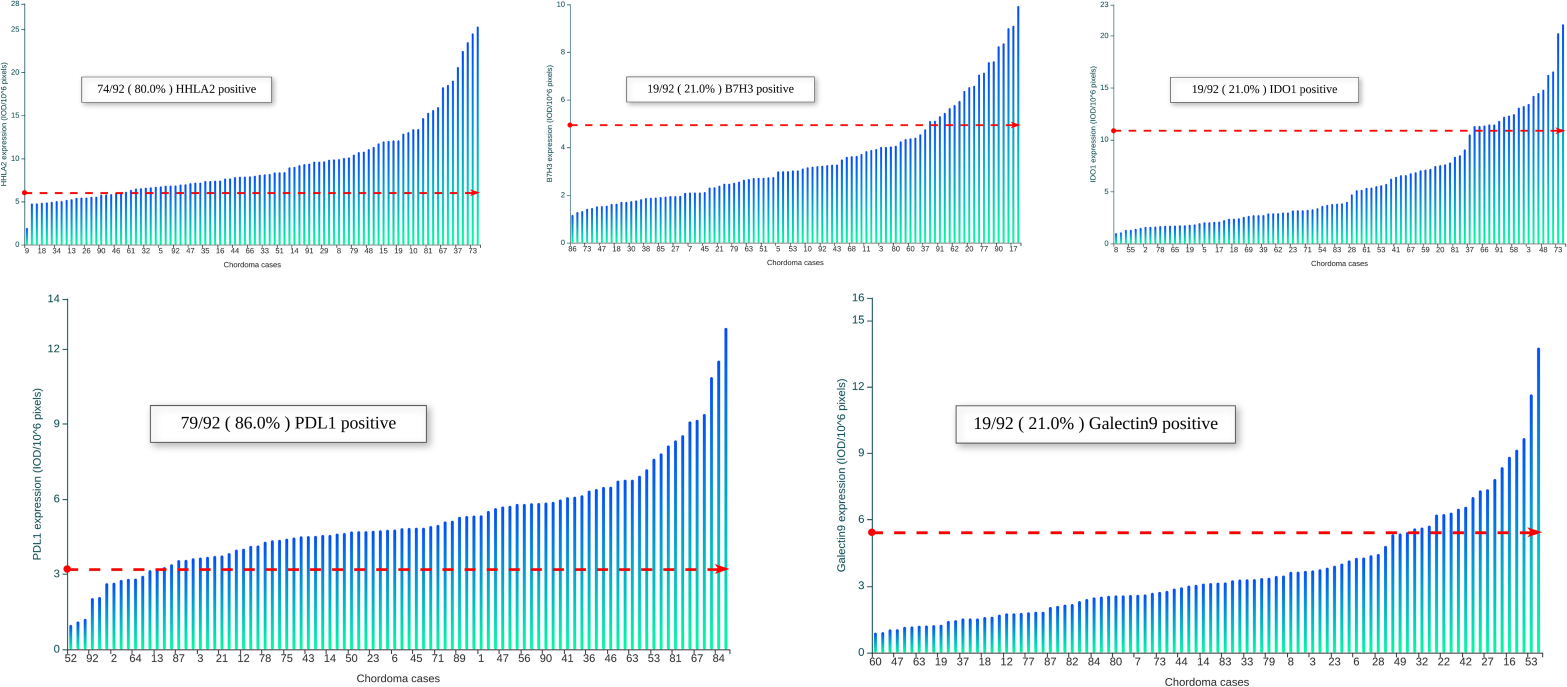
Distribution of HHLA2, B7H3, IDO-1, PD-L1 and Galectin-9 in 92 spinal chordoma samples. Scores are expressed as arbitrary units of fluorescence, and the dashed red line indicates the signal detection threshold determined as described in the methods section. The proportion of cases with detectable target signals is indicated within each chart.

Representative images showing various expression levels (specifically including negative, positive and strongly positive expression) of five QIF markers in chordoma tissues are depicted in **Figure 3**. PD-L1 and B7H3 expression had cytoplasmic and membranous staining, while HHLA2 and Galectin-9 expression showed predominant membranous staining. Unexpectedly, IDO-1 expression displayed a predominant perinuclear staining pattern. The average QIF scores of PD-L1, HHLA2, B7H3, IDO-1 and Galectin-9 were significantly higher in the stromal compartment than in the tumor subregion (**Figure 4**). However, 24, 6, 12, 21 and 6 patients displayed higher HHLA2, B7H3, IDO-1, PD-L1 and galectin-9 expression in the tumor subregion, respectively. According to the detection threshold described above, we found that tumoral HHLA2 and PD-L1 were positive in 80.0% (74/92) and 86.0% (79/92) of cases (**Figure 5**), respectively. In contrast, B7H3, IDO-1 and Galectin-9 positivity on tumor cells was only seen in 21.0% (19/92) of cases (**Figure 5**), although their expression was predominantly positive in the stromal area (**Supplemental Figure 2**).

**Figure 6:**
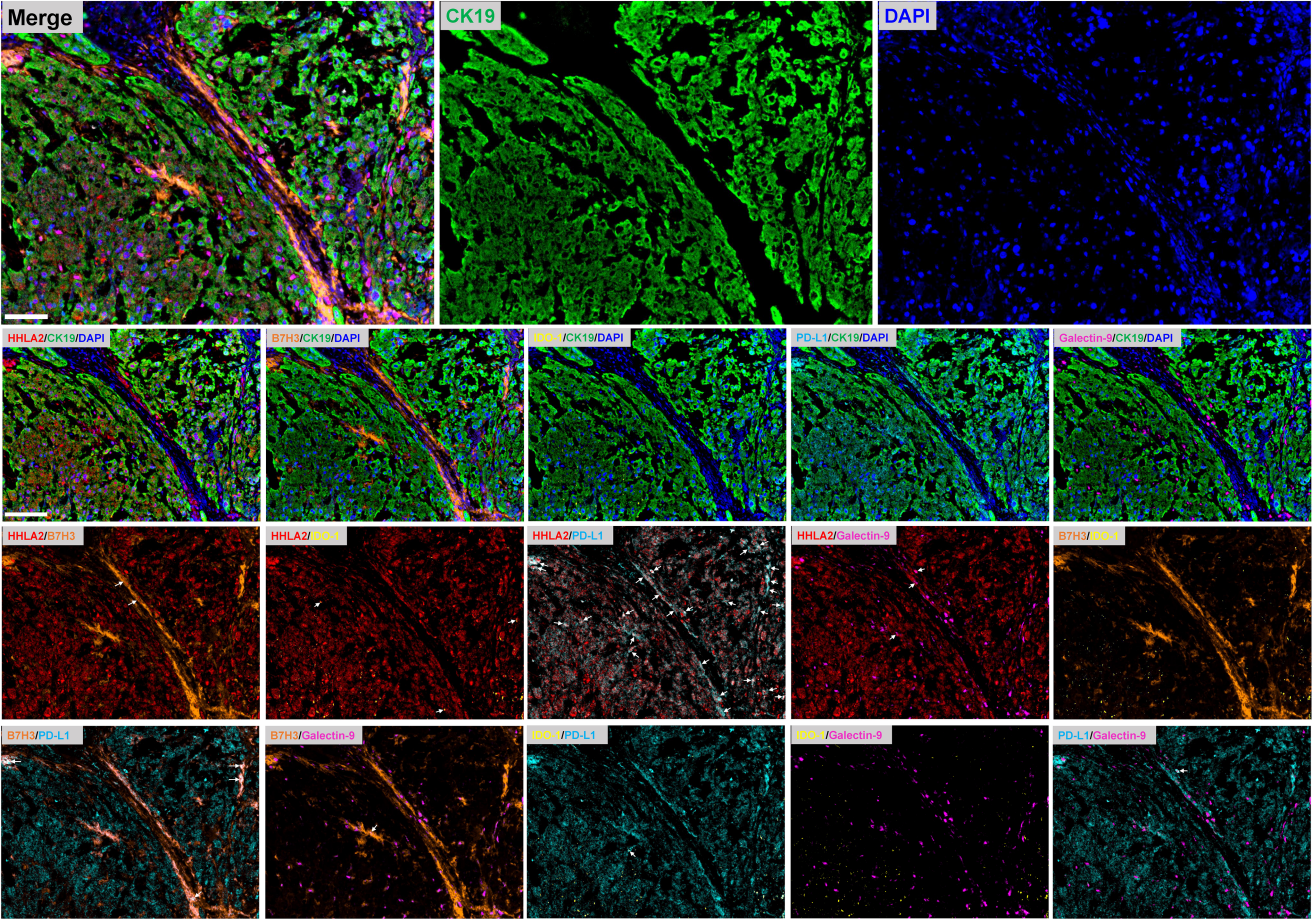
Representative fluorescence pictures show coexpression between immune checkpoints in human spinal chordoma. The white arrows indicate the coexpression of immune markers. The target protein is indicated in different fluorescence channels, and tumor cells are highlighted with CK-19. Nuclei are stained with DAPI. Scale bar = 100 μm.

**Figure 7:**
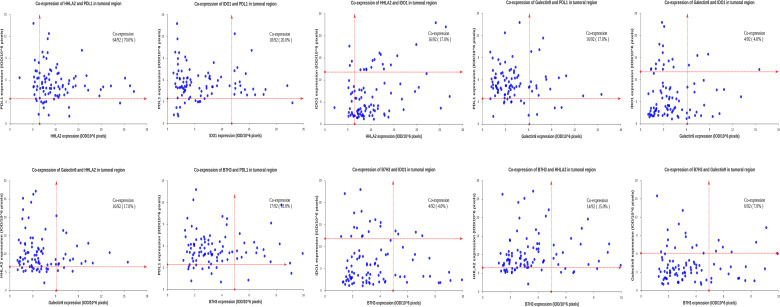
Coexpression of HHLA2, B7H3, IDO-1, PD-L1 and Galectin-9 in the tumor compartment of spinal chordoma samples. Scores are expressed as arbitrary units of fluorescence, and the dashed red line indicates the signal detection threshold determined as described in the methods section. The proportion of coexpressed cases is indicated within each chart.

We then analyzed the coexpression of the QIF markers in the tumor compartment, as well as the coexpression of B7H3, IDO-1 and Galectin-9 in the stromal compartment due to their high positivity rate in this subarea. Marker coexpression was defined when both proteins had positive staining in the same cells in the analyzed subregion; otherwise, no coexpression was recorded. We observed a high coexpression rate (64/92) for tumoral HHLA2 and PD-L1 in chordoma tissues, whereas other markers were infrequently coexpressed in this region (**Figures 6** and **7**). However, in the stromal compartment, B7H3, IDO-1 and Galectin-9 had remarkable coexpression (**Supplemental Figure 3**).

### Association of QIF Markers With Clinicopathological Features

**Figure 8:**
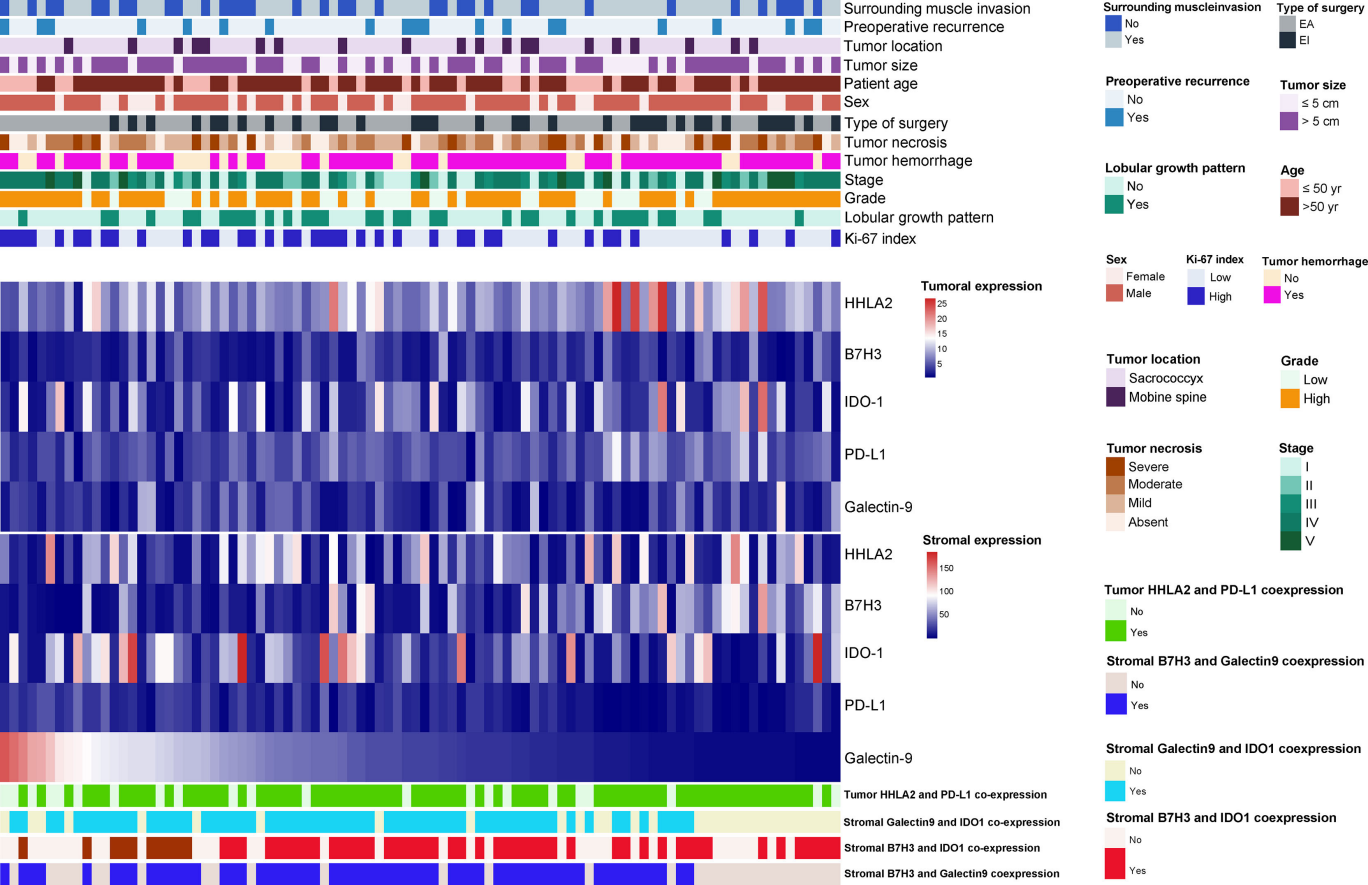
The heatmap shows the distribution of clinicopathological characteristics, quantitative immunofluorescence markers (including HHLA2, B7H3, IDO-1, PD-L1 and Galectin-9) expression and coexpression status among 92 spinal chordoma patients.

The relationships between the expression levels of the QIF markers and clinicopathological characteristics are displayed in **Figure 8** and **Supplementary Tables 3–5**. Patients with elevated tumoral HHLA2 expression or stromal B7H3 expression were more likely to receive EI resection of the tumor lesions. In contrast, a deceased stromal PD-L1 level was related to tumor hemorrhage, surrounding muscle invasion by tumors and EA surgical resection. We also found that tumors invading surrounding muscle tissues were significantly associated with high stromal expression of IDO-1. A similar positive correlation was also seen for stromal B7H3 expression and the tumor Ki-67 index. In addition, stromal Galctin-9 expression was related to the tumor Ki-67 proliferation index and EA resection of tumors. No significant correlation was detected between the QIF markers and other clinicopathologic variables studied, including tumor size and location. However, it should be noted that as no metastatic chordomas were included in this study, we were not able to analyze the association of these markers with tumor metastasis.

In terms of QIF marker coexpression, we discovered that male patients were more likely to have tumoral HHLA2/PD-L1 coexpression, which was also associated with EI resection of tumors. Similarly, stromal B7H3 and Galectin-9 coexpression was correlated with tumor necrosis and surrounding muscle invasion of tumors. In addition, our results also showed that stromal Galectin-9/IDO-1 coexpression was positively linked with lobular pattern growth.

### Association of QIF Markers With Microenvironmental Immune Parameters

Quantitative densities and representative images of TIL subtypes in the tumor and stroma are presented in **Supplemental Figures 4–6**. In the tumor subregion, PD-1-positive lymphocytes had the highest density, while CD20^+^ TIL infiltration was most abundant in the stromal compartment. In contrast, CD8^+^ T cells displayed the lowest infiltration level in both tumor and stromal subareas, indicating a suppressive microenvironment in chordoma tissues. **Supplemental Figure 7** and **Supplementary Table 6** show the association between QIF markers and microenvironmental TIL densities. Our study indicated that stromal PD-L1 and Galectin-9 levels were positively correlated with CD8^+^ TIL density in both the tumor and stromal regions. However, an inverse relationship between intratumoral or stromal PD-1^+^ TIL levels and stromal PD-L1 and Galectin-9 expression was observed. The number of stromal CD8^+^ TILs negatively correlated with tumoral HHLA2 expression, while PD-1^+^ TIL density showed a positive association with stromal B7H3 expression as well as the tumoral expression of HHLA2. Moreover, we also observed significant correlations between several QIF markers studied (**Supplemental Figure 7** and **Supplementary Table 6**).

### Association of QIF Markers With Survival

#### LRFS

**Figure 9:**
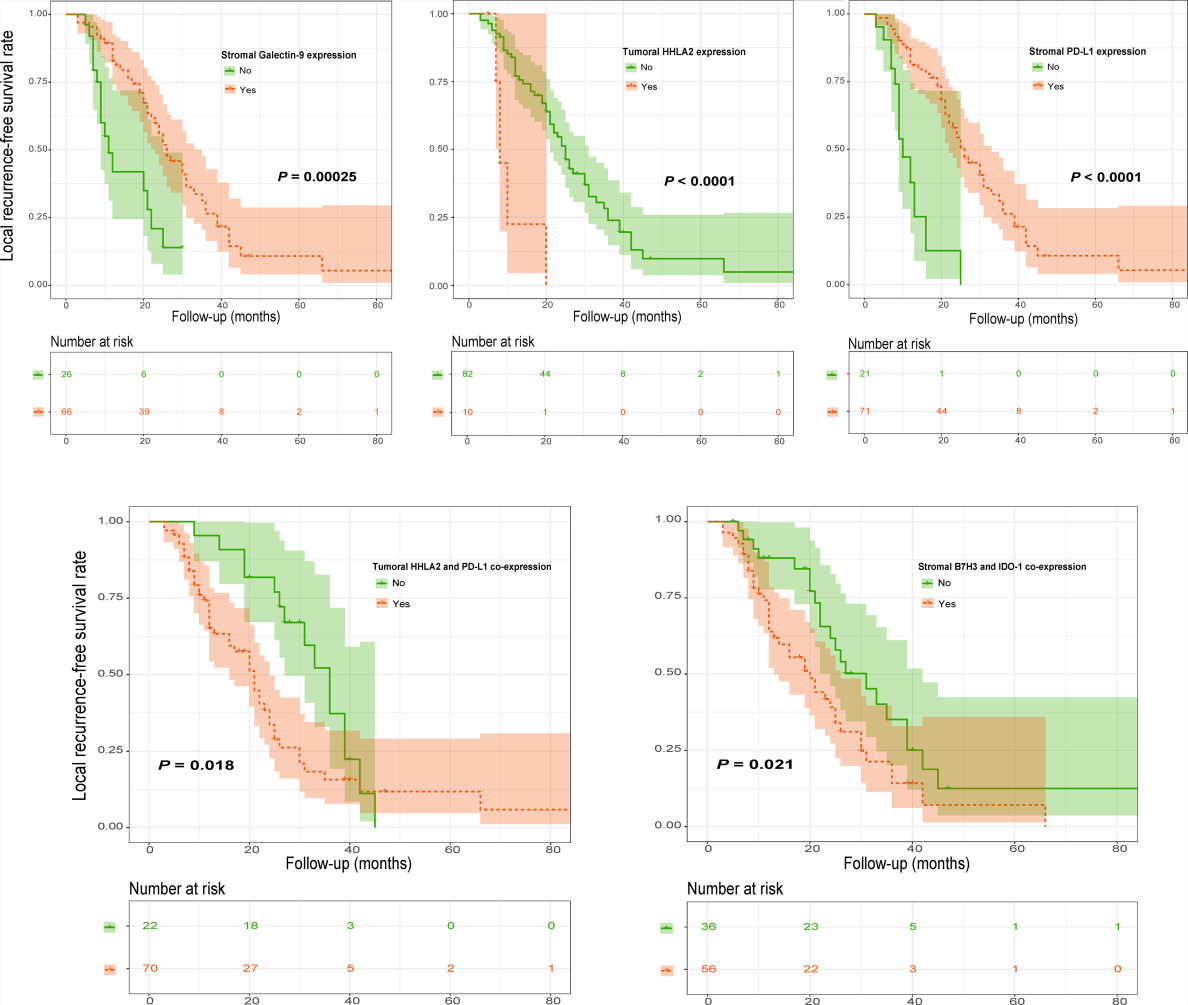
Kaplan–Meier curves of LRFS of spinal chordoma patients stratified by marker expression and coexpression status. LRFS, local recurrence-free survival.

**Figure 10:**
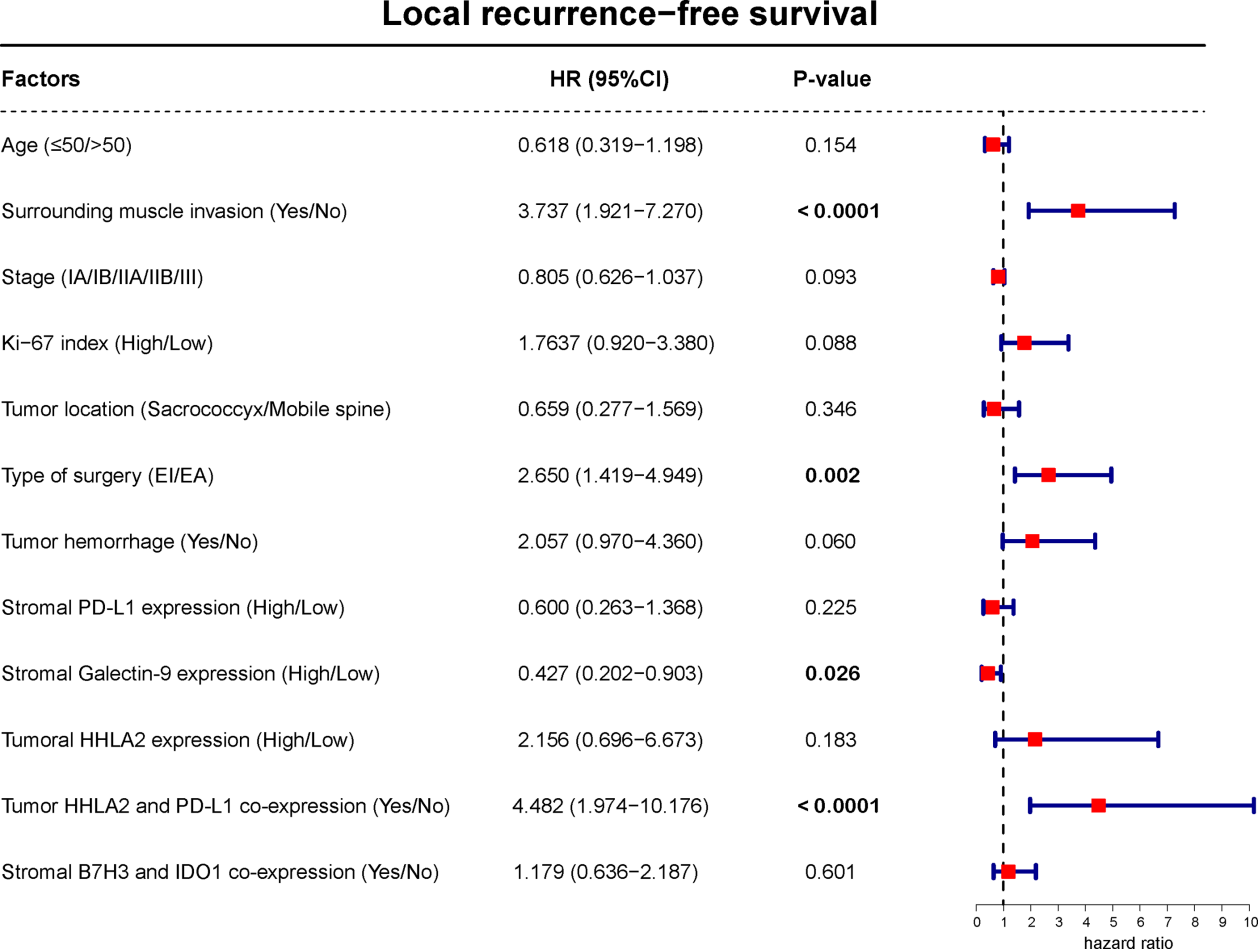
Multivariate Cox regression model including factors that were significant in univariate analysis for LRFS of spinal chordoma patients. The boxes indicate the hazard ratio, and the horizontal lines represent the 95% CI. Factors found to independently predict LRFS had *P* values in bold (less than 0.05). LRFS, local recurrence-free survival.

Kaplan–Meier analysis showed that high stromal PD-L1 or Galectin-9 levels were significantly related to better LFRS (**Figure 9** and **Supplementary Table 7**). However, high HHLA2 expression on tumor cells was significantly associated with shorter LFRS (**Figure 9**). In addition, our analysis also found that tumoral HHLA2 and PD-L1 coexpression significantly influenced LRFS (**Figure 9**). Similar observations were also observed for patient LRFS and stromal B7H3 and IDO-1 coexpression (**Figure 9**). However, tumoral PD-L1 expression (**Supplementary Table 7**) and recurrence status on admission (χ^2^ = 1.435, *P* = 0.231 by log-rank test) were not significantly associated with LRFS. Further multivariate Cox model confirmed stromal Galectin-9 expression as well as tumoral HHLA2/PD-L1 coexpression as independent predictors of LRFS (**Figure 10**).

#### OS

**Figure 11:**
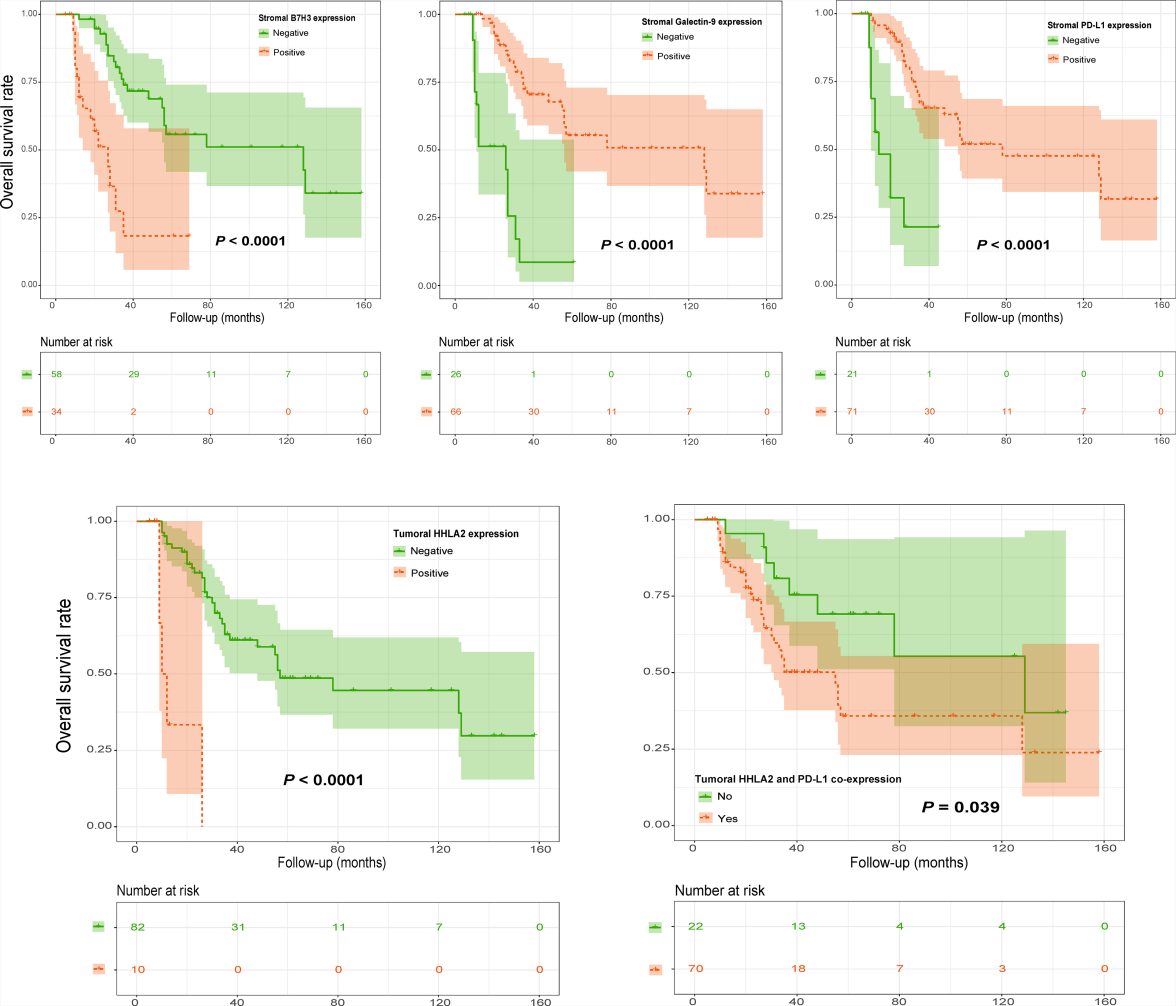
Kaplan–Meier curves of OS of spinal chordoma patients stratified by marker expression and coexpression status. OS, overall survival.

**Figure 12:**
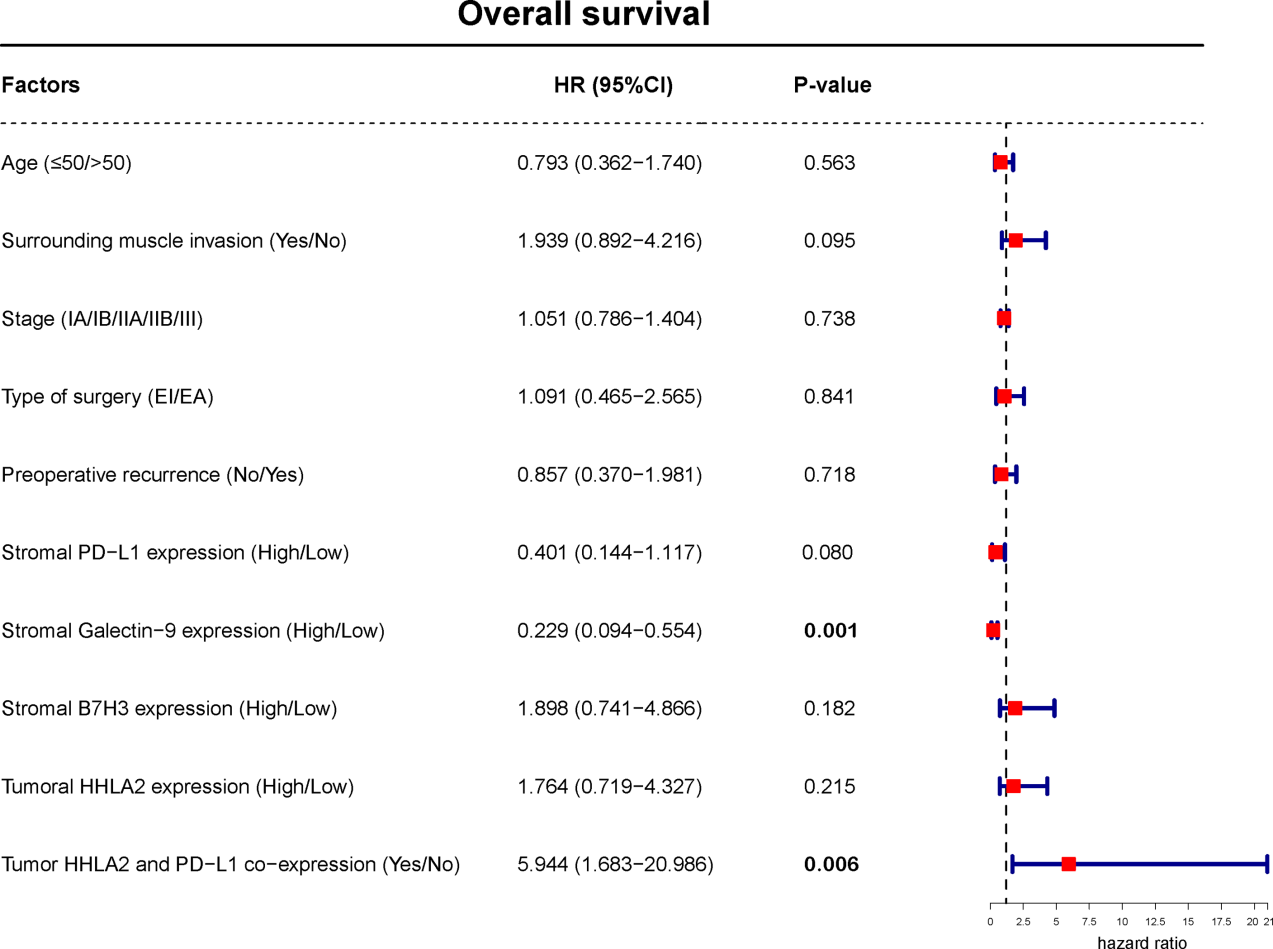
Multivariate Cox regression model including factors that were significant in univariate analysis for OS of spinal chordoma patients. The boxes indicate the hazard ratio, and the horizontal lines represent the 95% CI. Factors found to independently predict LRFS had *P* values in bold (less than 0.05). OS, overall survival.

Analyzing OS, Kaplan–Meier curves revealed that high tumoral HHLA2 expression or stromal B7H3 expression was significantly associated with worse survival (**Figure 11** and **Supplementary Table 8**). In contrast, this analysis indicated that stromal PD-L1 and galectin-9 levels had an inverse association with patient OS (**Figure 11**). Similarly, tumoral HHLA2 and PD-L1 coexpression was negatively associated with OS (**Figure 10**), which remained significant after multivariate adjustment (**Figure 12**). In addition, stromal Galectin-9 expression was also found to be independently predictive of OS (**Figure 12**). However, tumoral PD-L1 expression (**Supplementary Table 8**) and tumor recurrence on admission (χ^2^ = 0.275, *P* = 0.600 by log-rank test) failed to significantly influence OS.

## Discussion

In this study, we objectively analyzed the expression and coexpression of PD-L1, HHLA2, B7H3, IDO-1 and galectin-9 in spinal chordoma tissues and investigated the clinical impact of these immune checkpoints. We found a high positive rate of PD-L1 and HHLA2 proteins in the tumor subarea, whereas other markers had predominantly stromal expression. Coexpression of these QIF markers in the tumor compartment was scarcely detected except for PD-L1 and HHLA2. Further analyses showed that tumoral HHLA2 levels and stromal expression of several QIF markers (including PD-L1, B7H3 and Galectin-9) were associated with tumor phenotype, microenvironmental immunity level and clinical outcomes. Importantly, HHLA2 and PD-L1 coexpression on tumor cells independently predicted both worse LRFS and OS. These data provide a better understanding of the immunosuppressive mechanism in chordoma and may be useful for the development of combination or novel immunotherapy approaches aiming to improve patient survival.

We found that the HHLA2 protein was widely expressed in chordoma tissues and that tumoral HHLA2 levels significantly affected the survival of patients. These results were consistent with previous reports showing that HHLA2 was overexpressed in a variety of cancer cells (25, 36, 37) and could also independently portend patient outcomes in clear cell renal cell carcinoma and intrahepatic cholangiocarcinoma (25, 38). The precise mechanism underlying the relationship between tumoral HHLA2 expression and chordoma prognosis remains unknown. Published data have certified that HHLA2 mediates tumor escape from host immune attack by suppressing proliferation and cytokine production of both CD4^+^ and CD8^+^ T cells to promote disease progression by binding to TMIGD2 (36, 39). Similarly, we found that tumor HHLA2 expression was related to suppressive antitumor immunity (specifically including reduced CD8^+^ TIL density and increased PD-1^+^ T cell levels) in the chordoma microenvironment. Altogether, whether HHLA2 affects chordoma biology and patient outcome through TMIGD2 signaling or other molecular pathways to inhibit antitumor immune responses deserves further confirmation.

In addition, our study showed that tumoral HHLA2/PD-L1 coexpression was observed in most chordoma cases and could also independently predict prognosis, which was in line with the findings in clear cell renal cell carcinoma (38). These data may have implications for the use of immunotherapy combinations to improve clinical outcomes. Patients with the HHLA2^+^/PD-L1^+^ tumor phenotype may likely benefit from the dual blockade of PD-L1 and HHLA2 by immune-based drugs. Notably, we also disclosed that B7H3, IDO-1 and Galetin-9 showed limited coexpression with each other or with PD-L1, suggesting an independent biological function of these proteins in chordoma, similar to previous observations (27, 40). These findings hint at the possibility that only monotherapy with PD-1/PD-L1 inhibitors may not be able to obtain acceptable efficacy for patients. Future combinatorial therapy targeting more than one immune checkpoint signaling pathway may be a promising strategy to treat this disease.

Another major finding of our study was that stromal B7H3 expression in chordoma tissues was positively associated with the tumor Ki-67 index, a high density of PD-1^+^ TIL infiltration and poor prognosis. Similar to our data, previous studies reported that stromal B7H3 was closely correlated with an aggressive tumor phenotype, limited immune activation and worse prognosis in epithelial ovarian cancer (41). Researchers have demonstrated that cancer-associated fibroblasts (CAFs), as the main component of the stromal structure, can promote tumor growth and metastasis *via* various mechanisms, including contributing to an immunosuppressive microenvironment (42–44). Furthermore, it has been suggested that B7H3 could be highly expressed by microenvironmental CAFs (41). Considering that chordoma is histologically characterized by a rich myxoid stroma (45), these data strongly indicate that CAFs expressing B7H3 induce an inhibitory immune microenvironment to promote chordoma progression, thereby leading to adverse patient outcomes. This conjecture can be further supported by recent studies corroborating the prognostic significance of stromal content and immune cell infiltrates in spinal chordoma (29, 34, 45, 46). Our subsequent studies will further characterize the CAF profile and evaluate their association with B7H3 expression as well as the immune features in chordoma tissues.

It has been proven that Galectin-9 expression is closely correlated with the growth and metastasis of many solid cancers by inducing apoptosis of specific T cell subpopulations to mediate tumor immune escape (28, 47). In contrast, our analyses revealed that high stromal Galectin-9 or PD-L1 expression in chordoma was associated with less aggressive tumor features and accurately reflected better prognosis. In agreement with these outcomes, a recent report identified stromal galectin-9 as a favorable prognostic biomarker in lung cancer (48). A similar result was also described in small cell lung cancer, claiming that high galectin-9 expression on TILs indicated better LRFS in patients (49). Regarding stromal PD-L1 expression, prior studies have already suggested its positive prognostic implication in several malignant tumors, including chordoma (34, 50). Our current study provided further evidence for these findings. Previous reports have suggested that the expression level of stromal Galectin-9 represents preexisting antitumor immunity in the milieu (47). Our further analyses showed that stromal Galectin-9 or PD-L1 expression significantly correlated with an enhanced immune response, including increased CD8^+^ T cells and reduced PD-1^+^ TILs. It has been reported that CD8^+^ TIL density is linked to less invasive tumor features and favorable clinical outcomes in chordoma, while PD-1^+^ TILs exhibit poor clinicopathologic implications (18). Taken together, these data imply that stromal Galectin-9 or PD-L1 may influence chordoma phenotype and prognosis by promoting microenvironmental immune activation, although their specific regulatory networks require further investigation. Notably, in agreement with our previous observation (34), we still failed to define a significant correlation between tumoral PD-L1 level and patient survival, although this marker has been demonstrated as a robust predictor in various cancers (51). This inconsistency requires further confirmation and may be caused by the different tumor types studied.

### Limitations

Additional studies are required to further explore the mechanism of how QIF markers impact the clinical outcomes of patients. The expression and clinical role of several other immune checkpoint molecules (including VISTA, HVEM, ICOSL and GITRL) should also be evaluated in chordoma, and we did not perform this work due to restricted fluorescence channels available. Moreover, future works are needed to explore the expression pattern and biological functions of the respective ligands of PD-L1 and HHLA2 (namely, PD-1 and CD28) in chordoma tissues. In addition, our study did not identify relapsed tumors as a significant prognostic factor of chordoma outcome, which contradicts previous findings claiming that disease recurrence was closely related to poor patient survival in chordoma (8). This phenomenon may emerge due to the small number of recurrent patients included in this study, thus leading to low statistical power. Subsequent studies involving more recurrent patients are required to further test the prognostic value of tumor relapse in chordoma and compare the QIF outcomes with those of primary patients. Furthermore, future prospective studies with tumor progression as the endpoint of interest are necessary to describe chordoma outcome more accurately. Finally, our study is retrospective in nature, and future prospective studies involving large sample sizes are needed to validate our data.

## Conclusion

The present study objectively measured the tumoral and stromal expression of PD-L1, HHLA2, B7H3, IDO-1 and Galectin-9 in spinal chordoma tissues and analyzed their relationship with the clinical data of patients. We identified differential expression of the QIF markers between the tumor and stroma compartments. While PD-L1 and HHLA2 had remarkable coexpression in the tumor subarea, other markers were infrequently coexpressed in this region. In addition, tumoral HHLA2 expression and stromal levels of PD-L1, B7H3 and Galectin-9 could predict tumor features, the immune response in the microenvironment and patient outcomes. Importantly, tumoral HHLA2/PD-L1 coexpression in the tumor subregion independently influenced both LRFS and OS. These data may be useful for the development of combination or novel immune-based strategies to treat patients aiming to improve survival.

## Data Availability Statement

The original contributions presented in the study are included in the article/**Supplementary Material**. Further inquiries can be directed to the corresponding author.

## Ethics Statement

The study protocol was approved by the Institutional Review Board at The First Affiliated Hospital, University of South China, Hunan, P.R. China. Written informed consent was obtained from each patient for publication of this study. The patients/participants provided their written informed consent to participate in this study.

## Author Contributions

All authors participated in data acquisition. CX, WH, JL, CW and MXZ contributed to the conception and design of the study. YLC, HBF, MT, ZHO, TLZ, CX, WH and MXZ performed the data analysis and interpretation. CX, WH, GHL, YGY, NZY, CW and MXZ contributed to drafting and revision of the manuscript. All authors read and approved the final manuscript.
