# Corrigendum: Coexpression of HHLA2 and PD-L1 on tumor cells independently predicts the survival of spinal chordoma patients

**DOI:** 10.3389/fimmu.2023.1194064

**Published:** 2023-04-14

**Authors:** Chao Xia, Wei Huang, Yun-Liang Chen, Hai-Bin Fu, Ming Tang, Tao-Lan Zhang, Jing Li, Guo-Hua Lv, Yi-Guo Yan, Zhi-Hua Ouyang, Nvzhao Yao, Cheng Wang, Ming-Xiang Zou

**Affiliations:** ^1^ The First Affiliated Hospital, Health Management Center, Hengyang Medical School, University of South China, Hengyang, China; ^2^ Department of Spine Surgery, The First Affiliated Hospital, Hengyang Medical School, University of South China, Hengyang, China; ^3^ Shenzhen Audaque Data Technology Co., Ltd., Shenzhen, China; ^4^ Department of Spine Surgery, The Second Xiangya Hospital, Central South University, Changsha, China

**Keywords:** spinal chordoma, immune checkpoint molecules, quantitative immunofluorescence, HHLA2, PD-L1, tumor infiltrating lymphocytes

In the published article, there was an error in the author list, and Prof. Ming-Xiang Zou was erroneously denoted as the only corresponding author of this article. Prof. Cheng Wang should be denoted as the co-corresponding author for this paper. In addition, Prof. Cheng Wang and Prof. Ming-Xiang Zou should not have been marked with the † symbol. The corrected author list appears below.

Chao Xia^1,2†^, Wei Huang^1†^, Yun-Liang Chen^3^, Hai-Bin Fu^2^, Ming Tang^2^, Tao-Lan Zhang^2^, Jing Li ^4^, Guo-Hua Lv^4^, Yi-Guo Yan^2^, Zhi-Hua Ouyang^2^, Nvzhao Yao^2^, Cheng Wang^2*^ and Ming-Xiang Zou^2*^

The authors apologize for this error and state that this does not change the scientific conclusions of the article in any way. The original article has been updated.

