# Supplementary material for: Coexpression of HHLA2 and PD-L1 on Tumor Cells Independently Predicts the Survival of Spinal Chordoma Patients
Source: Front Immunol. 2022 Jan 25;12:797407. doi: 10.3389/fimmu.2021.797407 (PMC8824251; doi:10.3389/fimmu.2021.797407)
Supplement: Supplementary file 1 [file DataSheet_1.docx]

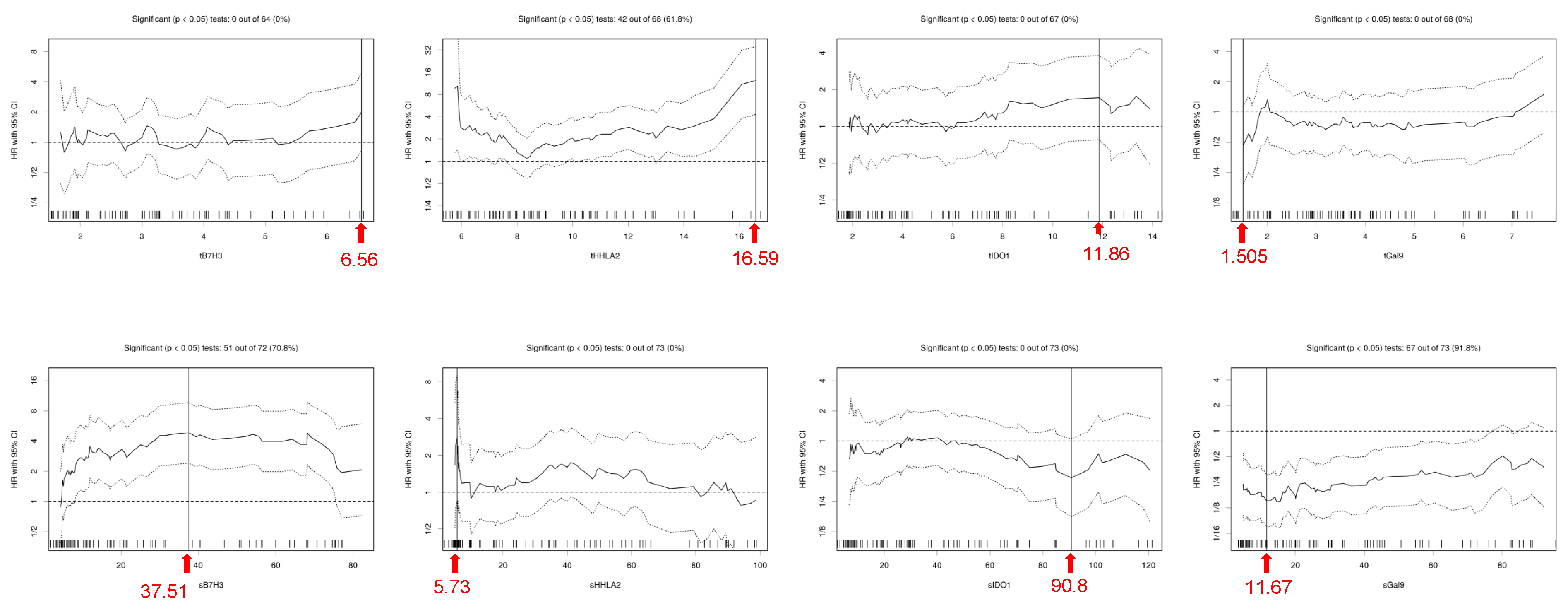


**Supplementary Figure 1.** Cutoff determination for the five markers in tumor and stromal subregions.

**
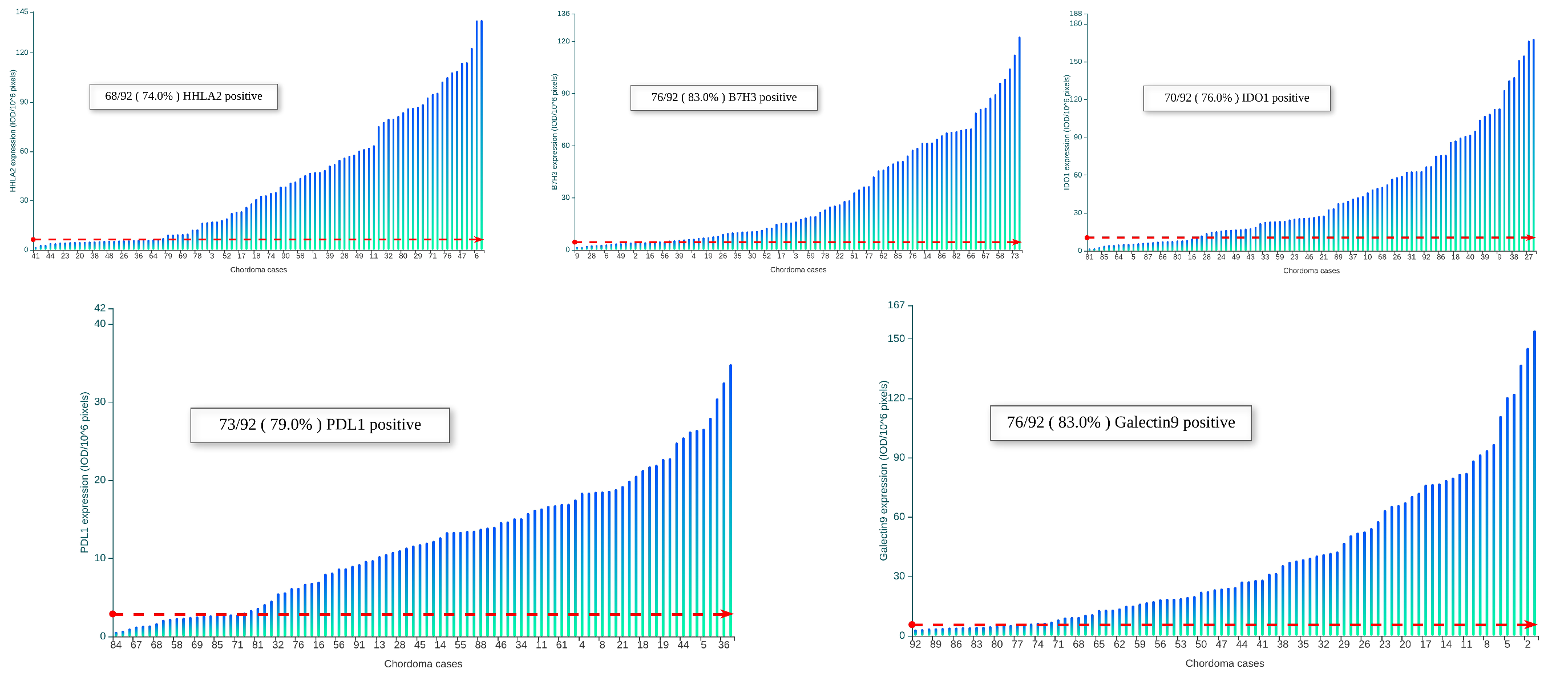
**

**Supplementary Figure 2.** Distribution of HHLA2, B7H3, IDO-1, PD-L1 and Galectin-9 in the stroma compartment. Scores are expressed as arbitrary units of fluorescence and the dashed red line indicates the signal detection threshold determined as described in the methods section. The proportion of cases with detectable target signal is indicated within each chart.

**
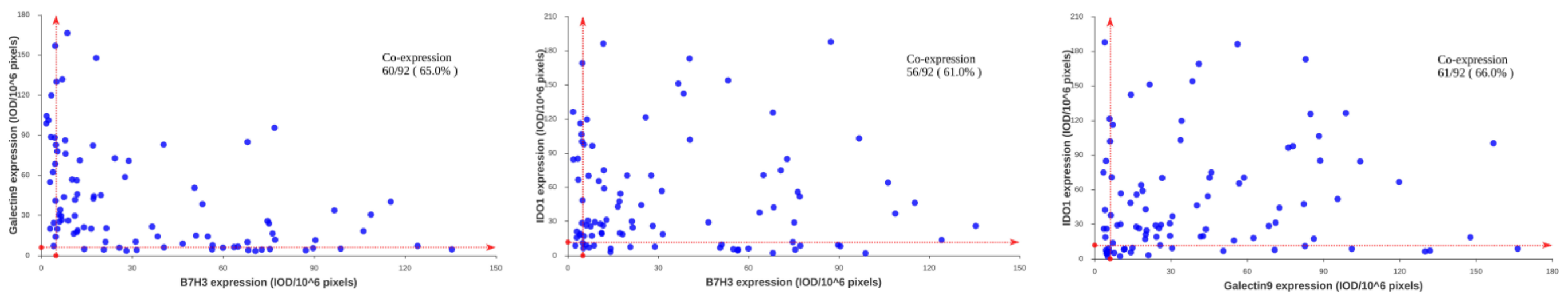
**

**Supplementary Figure 3.** Stromal co-expression of the markers based on the signal detection threshold (see methods section) are indicated within each chart.

**
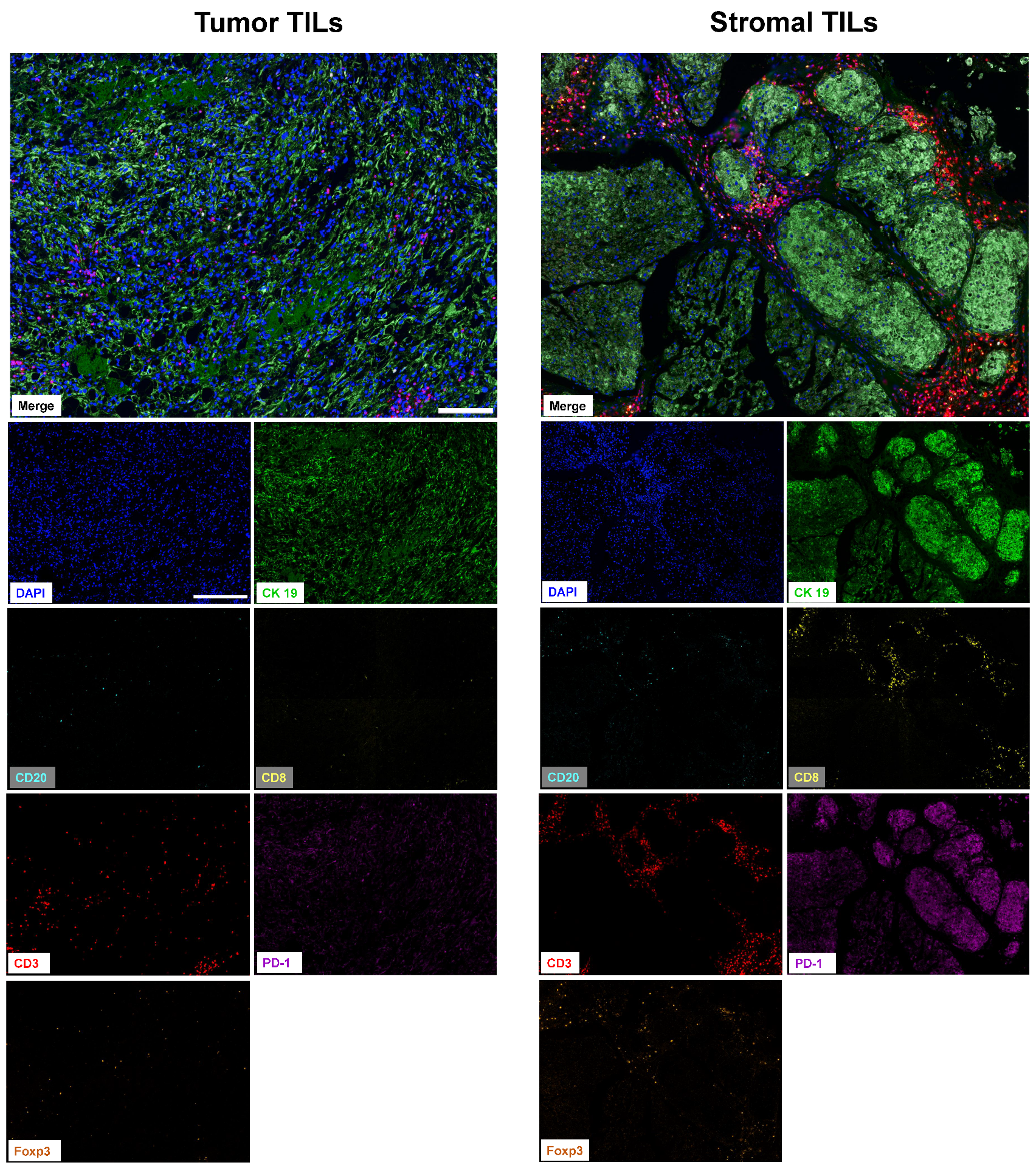
**

**Supplementary Figure 4.** Representative immunofluorescence images showing tumor-infiltrating lymphocytes level within tumor and stromal subregions of chordoma tissues. Scale bar = 100 um. Nuclei were stained with DAPI.


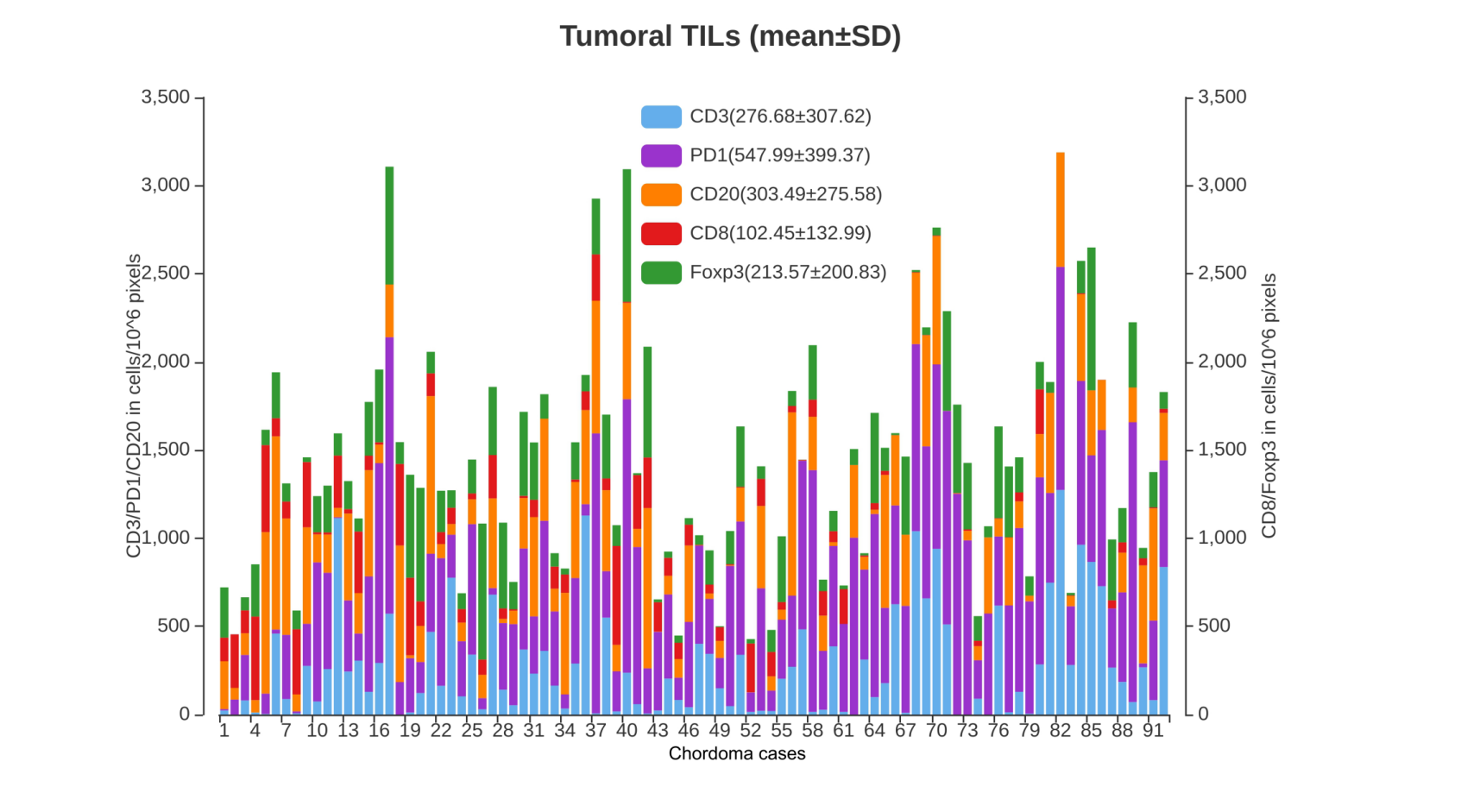


**Supplementary Figure 5.** Levels of TIL subpopulations in tumor. Distribution of tumoral CD3 (blue), CD8 (red), CD20 (orange) , PD1 (purple) and Foxp3 (green) QIF scores in human spinal chordoma.


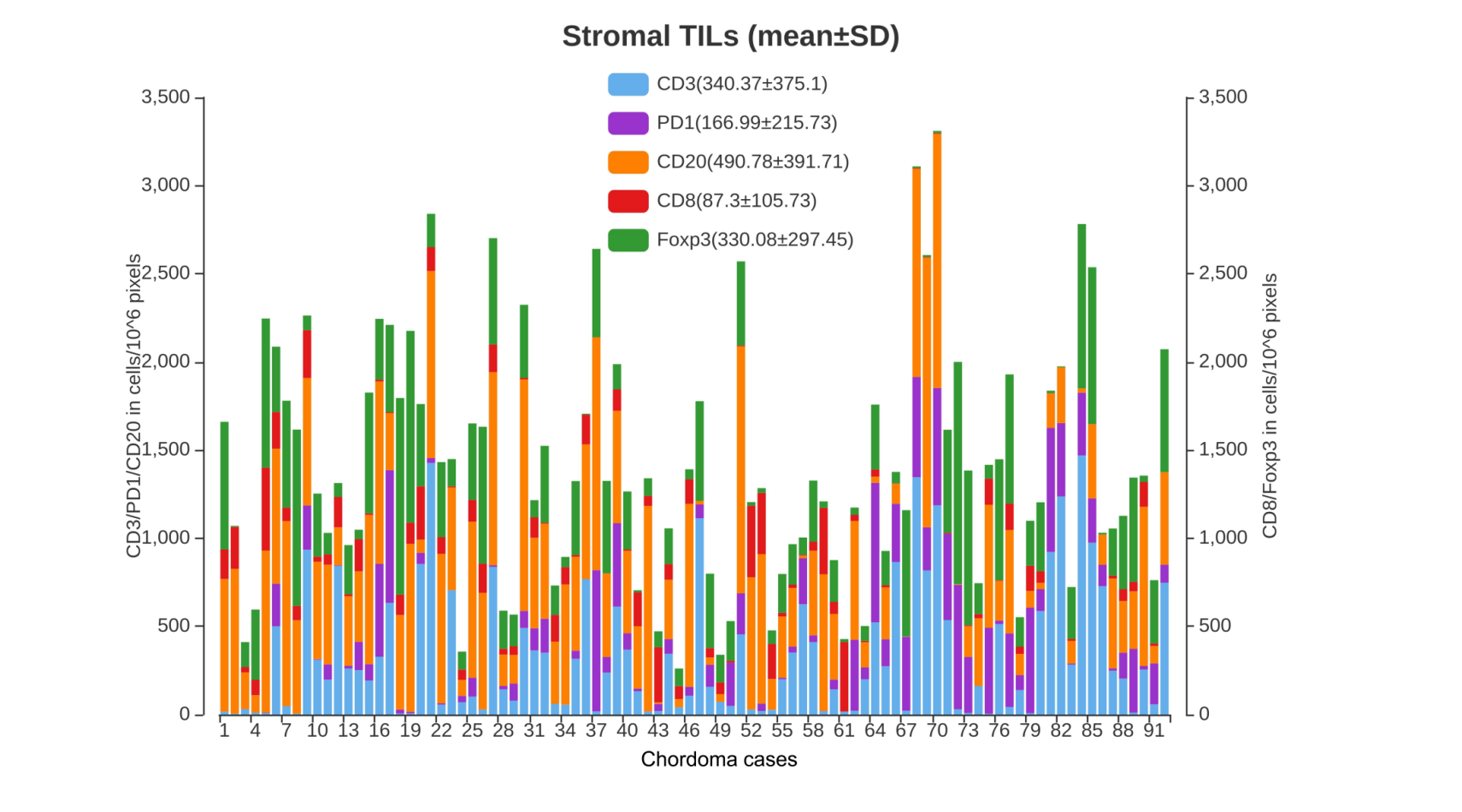


**Supplementary Figure 6.** Levels of TIL subpopulations in stroma. Distribution of stromal CD3 (blue), CD8 (red), CD20 (orange), PD1 (purple) and Foxp3 (green) QIF scores in human spinal chordoma.


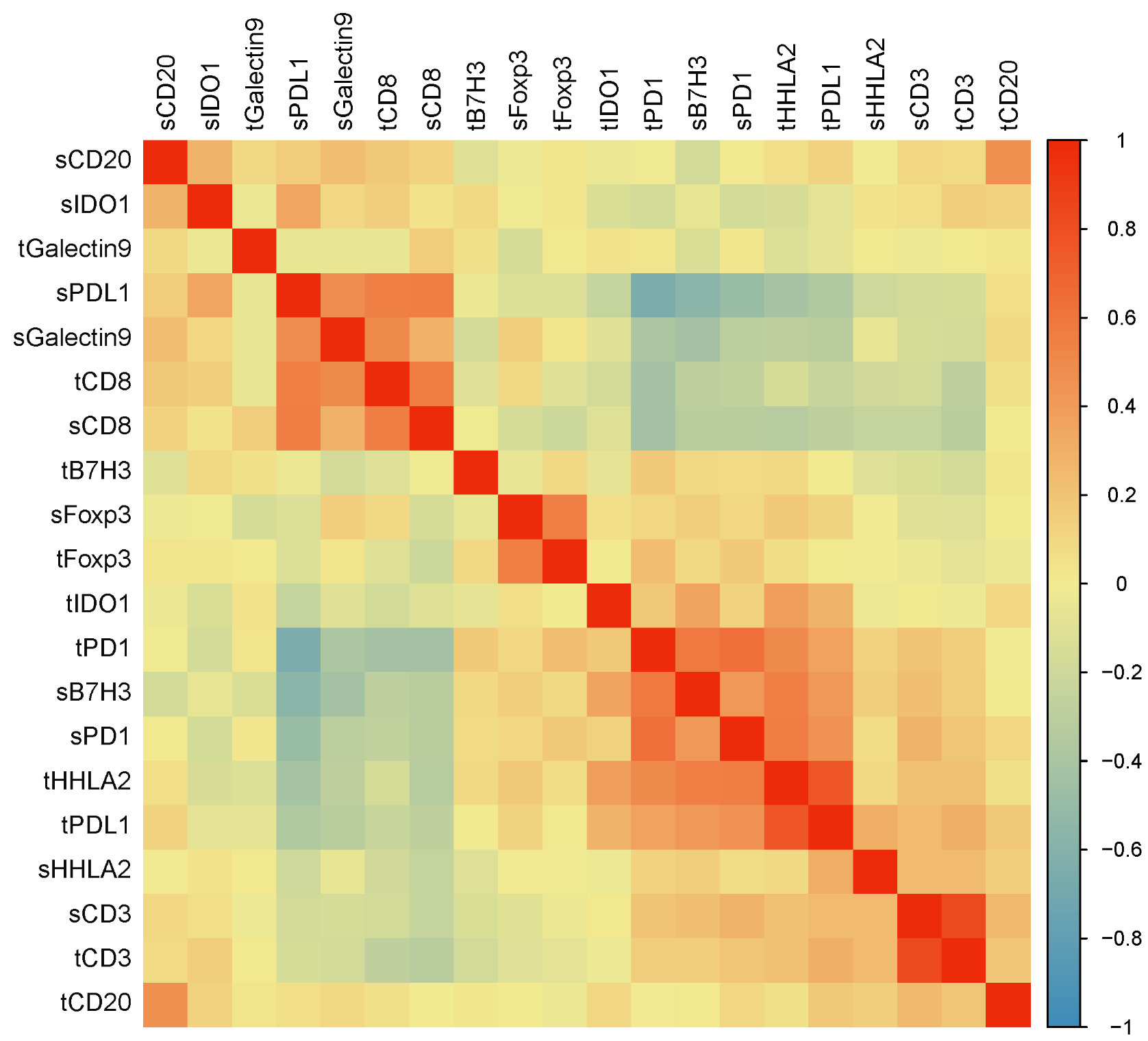


**Supplementary Figure 7.** The heatmap shows the correlation between the markers expression and TILs type among the chordoma samples. The lowercase letters ‘t’ and ‘s’ before each marker represent expression levels or densities of this marker (or marker-positive TILs) in the tumor and stromal compartment, respectively. Abbreviations: Gal9, Galectin-9, TILs, tumor-infiltrating lymphocytes.**Supplement Table 1. Detailed information on the isotype-specific primary antibodies**

| Antibody | Host Species | Description | Clone Number | Catalog Number | Company | Dilution |
| --- | --- | --- | --- | --- | --- | --- |
| Cytokeratin 19 | Mouse | Monoclonal | PT0087 | YM6167 | Immunoway | 1:5000 |
| CD3 | Rabbit | Monoclonal | SP7 | Kit-0003 | MXB Biotech | 1:10 |
| Foxp3 | Rabbit | Monoclonal | D2W8E | 98377 | CST | 1:500 |
| CD8 | Rabbit | Monoclonal | D8A8Y | 85336 | CST | 1:500 |
| CD20 | Mouse | Monoclonal | PT0029 | YM6149 | Immunoway | 1:5000 |
| PD-1 | Mouse | Monoclonal | ABT-PD1 | YM6208 | Immunoway | 1:10000 |
| Ki-67 | Rabbit | Monoclonal | SP6 | ab16667 | Abcam | 1:100 |
| B7H3 | Rabbit | Monoclonal | D9M2L | 14058 | CST | 1:300 |
| PD-L1 | Rabbit | Monoclonal | E1L3N | 13684 | CST | 1:300 |
| IDO-1 | Rabbit | Monoclonal | D5J4E | 86630 | CST | 1:1500 |
| HHLA2 | Rabbit | Polyclonal | No | Ab214327 | Abcam | 1:1500 |
| Galectin-9 | Mouse | Monoclonal | D9R4A | 54330 | CST | 1:3000 |

**Supplementary Table 2. Clinicopathological characteristics of 92 patients with spinal chordoma**

| Characteristic | Cohort (n = 92) |
| --- | --- |
| Age (years) |  |
| ≤ 50 | 30 (32.6%) |
| > 50 | 62 (67.4%) |
| Sex |  |
| Male | 67 (62.8%) |
| Female | 25 (27.2%) |
| Tumor size |  |
| ≤ 5 cm | 35 (38.0%) |
| > 5 cm | 57 (62.0%) |
| Tumor location |  |
| Sacrococcyx | 72 (78.3%) |
| Mobile spine | 20 (21.7%) |
| Surrounding muscle invasion |  |
| Yes | 57 (62.0%) |
| No | 35 (38.0%) |
| Preoperative recurrence |  |
| Yes | 20 (21.7%) |
| No | 72 (78.3%) |
| Grade |  |
| High | 61 (66.3%) |
| Low | 31 (33.7%) |
| Type of resection |  |
| EI | 34 (37.0%) |
| EA | 58 (63.0%) |
| Tumor hemorrhage |  |
| No | 25 (27.2%) |
| Yes | 67 (72.8%) |
| Tumor necrosis |  |
| Absent | 21 (22.8%) |
| Mild | 30 (32.6%) |
| Moderate | 24 (26.1%) |
| Severe | 17 (18.5%) |
| Ki-67 index |  |
| Low | 39 (42.4%) |
| High | 53 (57.6%) |
| Lobular growth pattern |  |
| No | 57 (62.0%) |
| Yes | 35 (38.0%) |
| Enneking staging |  |
| IA | 19 (20.7%) |
| IB | 11 (12.0%) |
| IIA | 11 (12.0%) |
| IIB | 41 (44.6%) |
| III | 10 (10.9%) |
| Follow-up duration (months) | 40.98 ± 35.52 |

Abbreviations: EI, Enneking inappropriate; EA, Enneking appropriate;

**Supplementary Table 3. Association between tumoral PD-L1, HHLA2, B7-H3, IDO-1 and Galectin-9 level and clinicopathological features of 92 spinal chordoma patients**

| Factors | Categories | Number of patients (n) | HHLA2 | | B7-H3 | | IDO-1 | | PD-L1 | | Galectin-9 | |
| --- | --- | --- | --- | --- | --- | --- | --- | --- | --- | --- | --- | --- |
|  |  |  | Mean level | *P*-value | Mean level | *P*-value | Mean level | *P*-value | Mean level | *P*-value | Mean level | *P*-value |
| Age (years) | ≤50 | 30 | 9.83 ± 5.32 | 0.646 | 3.43 ± 1.86 | 0.572 | 7.01 ± 5.11 | 0.523 | 5.78 ± 2.38 | 0.511 | 4.08 ± 2.38 | 0.850 |
|  | >50 | 62 | 10.35 ± 4.92 |  | 3.68 ± 2.10 |  | 6.28 ± 5.16 |  | 5.44 ± 2.20 |  | 4.20 ± 2.95 |  |
| Gender | Female | 25 | 8.517 ± 3.26 | 0.052 | 3.662 ± 2.28 | 0.853 | 7.262 ± 4.79 | 0.400 | 4.882 ± 1.682 | 0.081 | 5.087 ± 3.72 | **0.049** |
|  | Male | 67 | 10.80 ± 5.44 |  | 3.573 ± 1.93 |  | 6.236 ± 5.26 |  | 5.802 ± 2.393 |  | 3.818 ± 2.25 |  |
| Size (cm) | ≤5cm | 35 | 11.06 ± 9.64 | 0.189 | 3.70 ± 2.29 | 0.710 | 6.52 ± 5.10 | 0.992 | 6.04± 2.42 | 0.103 | 3.92 ± 2.71 | 0.510 |
|  | >5cm | 57 | 9.64 ± 4.22 |  | 3.54 ± 1.85 |  | 6.51 ± 5.19 |  | 5.25 ± 2.11 |  | 4.31 ± 2.81 |  |
| Tumor location | Sacrococcyx | 72 | 9.92 ± 4.79 | 0.354 | 3.53 ± 2.02 | 0.572 | 6.49 ± 5.40 | 0.940 | 5.37 ± 2.12 | 0.152 | 4.01 ± 2.66 | 0.331 |
|  | Mobile spine | 20 | 11.11 ± 5.86 |  | 3.83 ± 2.05 |  | 6.59 ± 4.14 |  | 6.19 ± 2.65 |  | 4.70 ± 3.13 |  |
| Type of resection | EA | 58 | 9.21 ± 4.25 | **0.015** | 3.491 ± 1.92 | 0.511 | 6.26 ± 4.74 | 0.536 | 5.254 ± 2.16 | 0.098 | 3.96 ± 2.08 | 0.359 |
|  | EI | 34 | 11.84 ± 5.84 |  | 3.78 ± 2.20 |  | 6.95 ± 5.78 |  | 6.06 ± 2.35 |  | 4.51 ±3.65 |  |
| Surrounding tissue invasion | No | 35 | \| 9.71 ± 4.19 \| \| --- \| | 0.485 | 3.46 ± 2.07 | 0.609 | 6.954 ± 5.62 | 0.522 | 5.25 ± 2.07 | 0.319 | 4.50 ± 3.01 | 0.366 |
|  | Yes | 57 | \| 10.47 ± 5.50 \| \| --- \| |  | 3.68 ± 2.00 |  | 6.245 ± 4.84 |  | 5.74 ± 2.36 |  | 3.96 ± 2.61 |  |
| Preoperative recurrence | No | 72 | 10.43 ± 5.14 | 0.367 | 3.81 ± 2.03 | 0.051 | 6.82 ± 5.26 | 0.285 | 5.51 ± 2.45 | 0.742 | 4.20 ± 2.71 | 0.792 |
|  | Yes | 20 | 9.28 ± 4.62 |  | 2.82 ± 1.84 |  | 5.42 ± 4.60 |  | 5.70 ± 1.37 |  | 4.02 ± 3.00 |  |
| Grade | Low | 31 | 10.93 ± 5.61 | 0.310 | 3.96 ± 2.08 | 0.222 | 6.77 ± 4.73 | 0.741 | 5.59 ± 2.41 | 0.902 | 3.98 ± 2.48 | 0.661 |
|  | High | 61 | 9.80 ± 4.71 |  | 3.41 ± 1.98 |  | 6.39 ± 5.36 |  | 5.53 ± 2.19 |  | 4.25 ± 2.91 |  |
| Enneking staging | IA | 19 | 10.52±6.08 | 0.332 | 4.22±2.29 | 0.269 | 6.11±4.30 | 0.473 | 5.55±2.65 | 0.851 | 4.88±2.81 | 0.451 |
|  | IB | 11 | 13.48±6.06 |  | 3.37±2.14 |  | 9.27±7.52 |  | 6.73±3.11 |  | 4.61±2.67 |  |
|  | IIA | 11 | 9.40±2.97 |  | 3.87±1.80 |  | 6.79±3.76 |  | 4.65±1.60 |  | 3.41±1.79 |  |
|  | IIB | 41 | 9.58±4.60 |  | 3.44±1.91 |  | 6.18±5.35 |  | 5.56±1.90 |  | 3.84±2.64 |  |
|  | III | 10 | 9.25±4.29 |  | 3.02±1.98 |  | 5.33±3.20 |  | 5.20±2.07 |  | 4.45±3.84 |  |
| Tumor hemorrhage | No | 25 | 9.47±5.22 | 0.414 | 3.69±2.21 | 0.780 | 6.05±4.54 | 0.597 | 5.35±2.42 | 0.601 | 4.17±2.05 | 0.990 |
|  | Yes | 67 | 10.44±5.22 |  | 3.56±1.96 |  | 6.69±5.35 |  | 5.63±2.20 |  | 4.16±3.00 |  |
| Tumor necrosis | Absent | 21 | 10.19 ± 6.08 | 0.867 | 3.22 ±1.39 | 0.925 | 6.58 ± 5.94 | 0.780 | 5.85 ± 2.48 | 0.988 | 4.31 ± 2.27 | 0.457 |
|  | Mild | 30 | 10.72 ± 5.27 |  | 3.62 ± 3.11 |  | 5.92 ± 4.37 |  | 5.45 ± 2.37 |  | 4.46 ± 2.83 |  |
|  | Moderate | 24 | 9.63 ± 3.35 |  | 3.97 ± 2.46 |  | 7.38 ± 5.38 |  | 5.47 ± 1.83 |  | 4.36 ± 3.63 |  |
|  | Severe | 17 | 10.01 ± 5.51 |  | 3.50 ± 1.90 |  | 6.26 ± 5.25 |  | 5.49 ± 2.45 |  | 3.18 ± 1.50 |  |
| Ki-67 | Low | 39 | 9.90±5.46 | 0.645 | 3.34±1.80 | 0.301 | 6.00±4.65 | 0.410 | 5.04±2.51 | 0.062 | 4.04±2.27 | 0.718 |
|  | High | 53 | 10.39±4.73 |  | 3.79±2.16 |  | 6.90±5.47 |  | 5.93±1.98 |  | 4.25±3.09 |  |
| Lobular pattern growth | No | 57 | 10.09±4.74 | 0.825 | 3.58±1.93 | 0.937 | 6.16±5.05 | 0.405 | 5.52±2.39 | 0.850 | 4.04±2.89 | 0.600 |
|  | Yes | 35 | 10.33±5.55 |  | 3.62±2.18 |  | 7.09±5.28 |  | 5.61±2.04 |  | 4.36±2.57 |  |

Bold values indicate *P* < 0.05. Abbreviations: EI, Enneking inappropriate; EA, Enneking appropriate.

**Supplementary Table 4. Association between stromal PD-L1, HHLA2, B7-H3, IDO-1 and Galectin-9 level and clinicopathological features of 92 spinal chordoma patients**

| Factors | Categories | Number of patients (n) | HHLA2 | | B7H3 | | IDO-1 | | PD-L1 | | Galctin-9 | |
| --- | --- | --- | --- | --- | --- | --- | --- | --- | --- | --- | --- | --- |
|  |  |  | Mean level | *P*-value | Mean level | *P*-value | Mean level | *P*-value | Mean level | *P*-value | Mean level | *P*-value |
| Age (years) | ≤50 | 30 | 43.96 ± 43.41 | 0.821 | 32.95 ± 33.31 | 0.687 | 44.87 ± 41.84 | 0.425 | 12.91 ± 8.36 | 0.257 | 49.83 ± 49.32 | 0.146 |
|  | >50 | 62 | 41.98 ± 37.17 |  | 36.06 ± 35.17 |  | 53.41 ± 50.46 |  | 15.41 ± 10.48 |  | 37.07 ± 33.16 |  |
| Gender | Female | 25 | 40.29 ± 43.92 | 0.729 | 26.54 ± 28.65 | 0.149 | 55.05 ± 53.79 | 0.590 | 16.08 ± 9.70 | 0.379 | 41.52 ± 32.27 | 0.967 |
|  | Male | 67 | 43.49 ± 37.44 |  | 38.22 ± 36.03 |  | 48.97 ± 45.63 |  | 14.03 ±9.94 |  | 41.12 ± 41.91 |  |
| Size (cm) | ≤5cm | 35 | 44.38 ± 41.34 | 0.737 | 33.58 ± 35.11 | 0.752 | 48.27 ± 45.95 | 0.713 | 14.62 ± 10.38 | 0.982 | 38.95 ± 39.65 | 0.666 |
|  | >5cm | 57 | 41.54 ± 37.97 |  | 35.94 ± 34.28 |  | 52.07 ± 49.18 |  | 14.57 ± 9.63 |  | 42.63 ± 39.45 |  |
| Tumor location | Sacrococcyx | 72 | 39.79 ± 37.03 | 0.189 | 35.15 ± 35.61 | 0.955 | 50.79 ± 46.80 | 0.951 | 14.68 ± 10.25 | 0.871 | 42.88 ± 41.72 | 0.449 |
|  | Mobile spine | 20 | 52.81 ± 45.32 |  | 34.66 ± 30.59 |  | 50.04 ± 52.34 |  | 14.27 ± 8.55 |  | 35.30 ± 29.40 |  |
| Type of resection | EA | 58 | 39.53 ± 38.92 | 0.325 | 27.22 ± 29.16 | 0.004 | 49.52 ± 46.55 | 0.774 | 16.99 ± 9.21 | **0.002** | 49.00 ± 43.72 | **0.013** |
|  | EI | 34 | 47.89 ± 39.38 |  | 48.38 ± 38.84 |  | 52.50 ± 50.41 |  | 10.49 ± 9.71 |  | 27.99 ± 26.13 |  |
| Surrounding tissue invasion | No | 35 | 49.77 ± 42.07 | 0.171 | 26.91 ± 34.85 | 0.076 | 69.88 ± 58.75 | **0.002** | 18.72 ± 9.88 | **0.001** | 48.47 ± 41.04 | 0.168 |
|  | Yes | 57 | 38.24 ± 36.83 |  | 40.03 ± 33.49 |  | 38.80 ± 35.16 |  | 12.05 ± 9.04 |  | 36.79 ± 37.96 |  |
| Preoperative recurrence | No | 72 | 42.83 ± 38.43 | 0.926 | 34.74 ± 33.62 | 0.874 | 54.20 ± 48.81 | 0.174 | 14.61 ± 9.91 | 0.973 | 40.45 ± 37.28 | 0.718 |
|  | Yes | 20 | 41.90 ±42.38 |  | 36.13 ± 38.08 |  | 37.74 ± 42.40 |  | 14.52 ± 9.95 |  | 44.07 ± 47.03 |  |
| Grade | Low | 31 | 42.63 ± 40.13 | 1.00 | 31.61 ± 33.68 | 0.498 | 54.29 ± 43.97 | 0.602 | 15.50 ± 10.36 | 0.534 | 35.17 ± 25.68 | 0.295 |
|  | High | 61 | 42.62 ± 38.88 |  | 36.79 ± 34.94 |  | 48.76 ± 49.82 |  | 14.13 ± 9.66 |  | 44.31 ± 44.61 |  |
| Enneking staging | IA | 19 | 44.08 ± 39.62 | 0.853 | 26.72±28.87 | 0.622 | 60.82±48.57 | 0.273 | 16.16±10.74 | 0.056 | 34.02±25.49 | 0.820 |
|  | IB | 11 | 53.22± 42.88 |  | 31.69±41.46 |  | 33.47±32.67 |  | 13.35±11.33 |  | 39.58±29.65 |  |
|  | IIA | 11 | 49.04± 38.50 |  | 34.90±35.14 |  | 76.76±67.56 |  | 15.90±8.04 |  | 31.67±27.53 |  |
|  | IIB | 41 | 36.67± 35.78 |  | 36.21± 35.71 |  | 44.83± 42.12 |  | 14.37±8.73 |  | 48.48±47.48 |  |
|  | III | 10 | 45.53± 48.04 |  | 49.92± 29.31 |  | 45.14± 48.42 |  | 12.43±12.92 |  | 37.53±42.84 |  |
| Tumor hemorrhage | No | 25 | 50.78±42.42 | 0.224 | 26.45±28.12 | 0.105 | 67.27±60.45 | 0.094 | 18.26±11.17 | **0.028** | 52.33±38.49 | 0.099 |
|  | Yes | 67 | 39.58±37.65 |  | 38.25±36.17 |  | 44.49±40.93 |  | 13.22±9.05 |  | 37.09±39.15 |  |
| Tumor necrosis | Absent | 21 | 53.75 ± 45.05 | 0.489 | 27.69 ± 32.85 | 0.731 | 60.55 ± 52.18 | 0.426 | 16.35 ± 11.18 | 0.875 | 47.12 ± 47.65 | 0.879 |
|  | Mild | 30 | 39.91 ± 40.20 |  | 32.77 ± 33.01 |  | 40.77 ± 38.55 |  | 13.99 ± 10.38 |  | 36.61 ± 31.42 |  |
|  | Moderate | 24 | 41.95 ±39.49 |  | 39.79 ± 33.82 |  | 58.10 ± 51.03 |  | 13.98 ± 7.32 |  | 43.05 ± 37.62 |  |
|  | Severe | 17 | 34.61 ± 27.12 |  | 41.44 ± 40.18 |  | 45.2 ± 52.00 |  | 14.36 ± 10.94 |  | 39.54 ± 45.30 |  |
| Ki-67 | Low | 39 | 40.67±41.08 | 0.683 | 25.12±31.57 | **0.017** | 50.92±50.74 | 0.960 | 16.42±10.72 | 0.128 | 52.60±44.17 | **0.017** |
|  | High | 53 | 44.06±37.88 |  | 42.34±34.88 |  | 50.41±45.93 |  | 13.24±9.06 |  | 32.87±33.41 |  |
| Lobular pattern growth | No | 57 | 44.29±40.18 | 0.604 | 39.11±35.41 | 0.149 | 51.01±48.19 | 0.923 | 15.43±9.79 | 0.300 | 43.61±43.54 | 0.429 |
|  | Yes | 35 | 39.90±37.65 |  | 28.41±32.16 |  | 50.00±47.73 |  | 13.22±9.98 |  | 37.37±31.57 |  |

Bold values indicate *P* < 0.05. Abbreviations: EI, Enneking inappropriate; EA, Enneking appropriate.

**Supplementary Table 5.** Association between markers co-expression status and clinicopathological features of 92 spinal chordoma patients

| Factors | Categories | Tumoral HHLA2 and tPDL1 co-expression | | | | Stromal B7H3 and Galectin-9 co-expression | | | | Stromal B7H3 and IDO-1  co-expression | | | | Stromal Galectin-9 and IDO-1 co-expression | | | |
| --- | --- | --- | --- | --- | --- | --- | --- | --- | --- | --- | --- | --- | --- | --- | --- | --- | --- |
|  |  | Positive | Negative | χ^2^ | *P*-value | Positive | Negative | χ^2^ | *P*-value | Positive | Negative | χ^2^ | *P*-value | Positive | Negative | χ^2^ | *P*-value |
| Age (years) | ≤50 | 20 | 10 | 2.171 | 0.192 | 23 | 7 | 2.572 | 0.161 | 17 | 13 | 0.330 | 0.650 | 21 | 9 | 0.272 | 0.646 |
|  | >50 | 50 | 12 |  |  | 37 | 25 |  |  | 39 | 23 |  |  | 40 | 22 |  |  |
| Gender | Female | 14 | 11 | 7.63 | **0.012** | 16 | 9 | 0.022 | 1.00 | 15 | 10 | 0.539 | 0.764 | 17 | 8 | 0.044 | 1.00 |
|  | Male | 56 | 11 |  |  | 44 | 23 |  |  | 41 | 26 |  |  | 44 | 23 |  |  |
| Size (cm) | ≤5cm | 28 | 7 | 0.475 | 0.617 | 25 | 10 | 0.961 | 0.373 | 23 | 12 | 0.557 | 0.514 | 26 | 9 | 1.611 | 0.258 |
|  | >5cm | 42 | 15 |  |  | 35 | 22 |  |  | 33 | 24 |  |  | 35 | 22 |  |  |
| Tumor location | Sacrococcyx | 54 | 18 | 0.215 | 0.867 | 45 | 27 | 1.078 | 0.427 | 44 | 28 | 0.008 | 1.00 | 47 | 25 | 0.156 | 0.793 |
|  | Mobile spine | 16 | 4 |  |  | 15 | 5 |  |  | 12 | 8 |  |  | 14 | 6 |  |  |
| Type of resection | EA | 39 | 19 | 6.749 | **0.011** | 37 | 21 | 0.140 | 0.822 | 31 | 27 | 3.629 | 0.077 | 40 | 18 | 0.497 | 0.501 |
|  | EI | 31 | 3 |  |  | 23 | 11 |  |  | 25 | 9 |  |  | 21 | 13 |  |  |
| Surrounding muscle invasion | No | 23 | 12 | 3.341 | 0..082 | 18 | 17 | 4.735 | **0.042** | 18 | 17 | 2.114 | 0.188 | 25 | 10 | 0.664 | 0.498 |
|  | Yes | 47 | 10 |  |  | 42 | 15 |  |  | 38 | 19 |  |  | 36 | 21 |  |  |
| Preoperative recurrence | No | 56 | 16 | 0.520 | 0.671 | 46 | 26 | 0.258 | 0.792 | 43 | 29 | 0.183 | 0.798 | 49 | 23 | 0.455 | 0.595 |
|  | Yes | 14 | 6 |  |  | 14 | 6 |  |  | 13 | 7 |  |  | 12 | 8 |  |  |
| Grade | Low | 25 | 6 |  | 1.00 | 24 | 7 | 3.069 | 0.106 | 22 | 9 | 2.002 | 0.181 | 25 | 6 | 4.304 | 0.061 |
|  | High | 45 | 16 |  |  | 36 | 25 |  |  | 34 | 27 |  |  | 36 | 25 |  |  |
| Enneking staging^a^ | IA | 15 | 4 | 2.627 | 0.585 | 13 | 6 | 4.245 | 0.376 | 12 | 7 | 5.341 | 0.258 | 16 | 3 | 7.565 | 0.098 |
|  | IB | 10 | 1 |  |  | 9 | 2 |  |  | 8 | 3 |  |  | 7 | 4 |  |  |
|  | IIA | 7 | 4 |  |  | 7 | 4 |  |  | 8 | 3 |  |  | 9 | 2 |  |  |
|  | IIB | 30 | 11 |  |  | 27 | 14 |  |  | 25 | 16 |  |  | 25 | 16 |  |  |
|  | III | 8 | 2 |  |  | 4 | 6 |  |  | 3 | 7 |  |  | 4 | 6 |  |  |
| Tumor hemorrhage | No | 19 | 6 | 0.00 | 1.00 | 17 | 8 | 0.117 | 0.809 | 15 | 10 | 0.011 | 1.00 | 19 | 6 | 1.44 | 0.322 |
|  | Yes | 51 | 16 |  |  | 43 | 24 |  |  | 41 | 26 |  |  | 42 | 25 |  |  |
| Tumor necrosis^a^ | Absent | 17 | 4 | 1.752 | 0.643 | 14 | 7 | 8.123 | **0.037** | 13 | 8 | 3.854 | 0.283 | 17 | 4 | 4.838 | 0.181 |
|  | Mild | 24 | 6 |  |  | 25 | 5 |  |  | 21 | 9 |  |  | 20 | 10 |  |  |
|  | Moderate | 18 | 6 |  |  | 13 | 11 |  |  | 15 | 9 |  |  | 16 | 8 |  |  |
|  | Severe | 11 | 6 |  |  | 8 | 9 |  |  | 7 | 10 |  |  | 8 | 9 |  |  |
| Ki-67 | Low | 27 | 12 | 1.749 | 0.221 | 24 | 15 | 0.404 | 0.658 | 19 | 20 | 4.197 | 0.053 | 28 | 11 | 0.913 | 0.379 |
|  | High | 43 | 10 |  |  | 36 | 17 |  |  | 37 | 16 |  |  | 33 | 20 |  |  |
| LGP | No | 42 | 15 | 0.475 | 0.617 | 33 | 24 | 3.542 | 0.073 | 31 | 26 | 2.644 | 0.126 | 33 | 24 | 4.743 | **0.041** |
|  | Yes | 28 | 7 |  |  | 27 | 8 |  |  | 25 | 10 |  |  | 28 | 7 |  |  |

Bold values indicate *P* < 0.05. EI, Enneking inappropriate; EA, Enneking appropriate; LGP, Lobular growth pattern. ^a^Analyses were performed with Wilcoxon's rank sum test.

**Supplementary Table 6 Correlation between tumoral and stromal expression of markers and TILs (n = 92)**

|  |  | tHHLA2 | sHHLA2 | tB7H3 | sB7H3 | tIDO1 | sIDO1 | tPDL1 | sPDL1 | tGal9 | sGal9 | tPD1 | sPD1 | tCD8 | sCD8 | tCD20 | sCD20 | tCD3 | sCD3 | tFoxp3 | sFoxp3 |
| --- | --- | --- | --- | --- | --- | --- | --- | --- | --- | --- | --- | --- | --- | --- | --- | --- | --- | --- | --- | --- | --- |
| tHHLA2 | r | 1 | 0.09 | 0.087 | 0.549** | 0.386** | -0.151 | -0.136 | -0.420** | -0.125 | -0.288** | 0.492** | 0.563** | -0.163 | -0.327** | 0.053 | 0.058 | 0.214* | 0.214* | -0.106 | 0.019 |
|  | p value |  | 0.394 | 0.408 | 0 | 0 | 0.15 | 0.197 | 0 | 0.235 | 0.005 | 0 | 0 | 0.121 | 0.001 | 0.614 | 0.586 | 0.04 | 0.04 | 0.316 | 0.856 |
| sHHLA2 | r | 0.09 | 1 | -0.122 | 0.136 | -0.027 | 0.036 | -0.084 | -0.201 | 0.011 | -0.062 | 0.12 | 0.065 | -0.188 | -0.251* | 0.139 | -0.009 | 0.241* | 0.243* | -0.231* | -0.364** |
|  | p value | 0.394 |  | 0.246 | 0.197 | 0.797 | 0.731 | 0.424 | 0.054 | 0.919 | 0.559 | 0.253 | 0.537 | 0.072 | 0.016 | 0.187 | 0.929 | 0.02 | 0.019 | 0.027 | 0 |
| tB7H3 | r | 0.087 | -0.122 | 1 | 0.089 | -0.067 | 0.09 | -0.025 | -0.04 | 0.053 | -0.181 | 0.175 | 0.078 | -0.121 | -0.021 | 0.022 | -0.11 | -0.178 | -0.142 | 0.085 | 0.005 |
|  | p value | 0.408 | 0.246 |  | 0.398 | 0.524 | 0.393 | 0.811 | 0.703 | 0.615 | 0.085 | 0.095 | 0.46 | 0.249 | 0.841 | 0.834 | 0.298 | 0.09 | 0.177 | 0.419 | 0.96 |
| sB7H3 | r | 0.549** | 0.136 | 0.089 | 1 | 0.359** | -0.057 | -0.021 | -0.556** | -0.145 | -0.429** | 0.580** | 0.422** | -0.287** | -0.324** | 0.01 | -0.18 | 0.164 | 0.222* | -0.153 | -0.133 |
|  | p value | 0 | 0.197 | 0.398 |  | 0 | 0.59 | 0.84 | 0 | 0.168 | 0 | 0 | 0 | 0.006 | 0.002 | 0.928 | 0.086 | 0.118 | 0.034 | 0.145 | 0.206 |
| tIDO1 | r | 0.386** | -0.027 | -0.067 | 0.359** | 1 | -0.145 | -0.058 | -.249* | 0.036 | -0.103 | 0.178 | 0.12 | -0.185 | -0.109 | 0.1 | -0.044 | -0.025 | 0.013 | 0.203 | 0.08 |
|  | p value | 0 | 0.797 | 0.524 | 0 |  | 0.169 | 0.585 | 0.017 | 0.735 | 0.331 | 0.09 | 0.256 | 0.078 | 0.299 | 0.342 | 0.677 | 0.812 | 0.905 | 0.052 | 0.447 |
| sIDO1 | r | -0.151 | 0.036 | 0.09 | -0.057 | -0.145 | 1 | 0.091 | 0.351** | -0.036 | 0.103 | -0.172 | -0.17 | 0.152 | 0.044 | 0.119 | 0.281** | 0.141 | 0.063 | -0.186 | -0.259* |
|  | p value | 0.15 | 0.731 | 0.393 | 0.59 | 0.169 |  | 0.387 | 0.001 | 0.731 | 0.329 | 0.101 | 0.105 | 0.149 | 0.677 | 0.257 | 0.007 | 0.181 | 0.553 | 0.076 | 0.013 |
| tPDL1 | r | -0.136 | -0.084 | -0.025 | -0.021 | -0.058 | 0.091 | 1 | 0.113 | -0.086 | 0.085 | -0.066 | -0.087 | 0.093 | 0.04 | -0.009 | 0.011 | -0.125 | -0.102 | 0.092 | 0.107 |
|  | p value | 0.197 | 0.424 | 0.811 | 0.84 | 0.585 | 0.387 |  | 0.282 | 0.418 | 0.419 | 0.534 | 0.408 | 0.378 | 0.703 | 0.932 | 0.919 | 0.237 | 0.332 | 0.384 | 0.312 |
| sPDL1 | r | -0.420** | -0.201 | -0.04 | -0.556** | -0.249* | 0.351** | 0.113 | 1 | -0.047 | 0.480** | -0.654** | -0.486** | 0.548** | 0.574** | 0.063 | 0.158 | -0.157 | -0.176 | 0.116 | 0.210* |
|  | p value | 0 | 0.054 | 0.703 | 0 | 0.017 | 0.001 | 0.282 |  | 0.658 | 0 | 0 | 0 | 0 | 0 | 0.553 | 0.134 | 0.134 | 0.094 | 0.27 | 0.045 |
| tGal9 | r | -0.125 | 0.011 | 0.053 | -0.145 | 0.036 | -0.036 | -0.086 | -0.047 | 1 | -0.048 | 0.027 | 0.018 | -0.054 | 0.156 | 0.025 | 0.091 | 0.012 | -0.033 | -0.022 | -0.039 |
|  | p value | 0.235 | 0.919 | 0.615 | 0.168 | 0.735 | 0.731 | 0.418 | 0.658 |  | 0.648 | 0.798 | 0.867 | 0.61 | 0.137 | 0.812 | 0.389 | 0.907 | 0.751 | 0.836 | 0.711 |
| sGal9 | r | -0.288** | -0.062 | -0.181 | -0.429** | -0.103 | 0.103 | 0.085 | 0.480** | -0.048 | 1 | -0.392** | -0.301** | 0.488** | 0.288** | 0.089 | 0.234* | -0.183 | -0.163 | 0.246* | 0.252* |
|  | p value | 0.005 | 0.559 | 0.085 | 0 | 0.331 | 0.329 | 0.419 | 0 | 0.648 |  | 0 | 0.004 | 0 | 0.005 | 0.398 | 0.025 | 0.081 | 0.12 | 0.018 | 0.015 |
| tPD1 | r | 0.492** | 0.12 | 0.175 | 0.580** | 0.178 | -0.172 | -0.066 | -0.654** | 0.027 | -0.392** | 1 | 0.626** | -0.426** | -0.427** | -0.005 | -0.016 | 0.137 | 0.196 | -0.278** | -0.151 |
|  | p value | 0 | 0.253 | 0.095 | 0 | 0.09 | 0.101 | 0.534 | 0 | 0.798 | 0 |  | 0 | 0 | 0 | 0.96 | 0.878 | 0.192 | 0.061 | 0.007 | 0.15 |
| sPD1 | r | 0.563** | 0.065 | 0.078 | 0.422** | 0.12 | -0.17 | -0.087 | -0.486** | 0.018 | -0.301** | 0.626** | 1 | -0.266* | -0.316** | 0.104 | 0.008 | 0.19 | 0.277** | -0.238* | -0.162 |
|  | p value | 0 | 0.537 | 0.46 | 0 | 0.256 | 0.105 | 0.408 | 0 | 0.867 | 0.004 | 0 |  | 0.01 | 0.002 | 0.324 | 0.938 | 0.07 | 0.008 | 0.022 | 0.123 |
| tCD8 | r | -0.163 | -0.188 | -0.121 | -0.287** | -0.185 | 0.152 | 0.093 | .548** | -0.054 | 0.488** | -0.426** | -0.266* | 1 | 0.557** | 0.049 | 0.175 | -0.283** | -0.181 | 0.202 | 0.443** |
|  | p value | 0.121 | 0.072 | 0.249 | 0.006 | 0.078 | 0.149 | 0.378 | 0 | 0.61 | 0 | 0 | 0.01 |  | 0 | 0.646 | 0.095 | 0.006 | 0.084 | 0.053 | 0 |
| sCD8 | r | -0.327** | -0.251* | -0.021 | -0.324** | -0.109 | 0.044 | 0.04 | 0.574** | 0.156 | 0.288** | -0.427** | -0.316** | 0.557** | 1 | 0.003 | 0.13 | -0.316** | -0.247* | 0.182 | 0.366** |
|  | p value | 0.001 | 0.016 | 0.841 | 0.002 | 0.299 | 0.677 | 0.703 | 0 | 0.137 | 0.005 | 0 | 0.002 | 0 |  | 0.981 | 0.218 | 0.002 | 0.017 | 0.083 | 0 |
| tCD20 | r | 0.053 | 0.139 | 0.022 | 0.01 | 0.1 | 0.119 | -0.009 | 0.063 | 0.025 | 0.089 | -0.005 | 0.104 | 0.049 | 0.003 | 1 | 0.463** | 0.19 | 0.251* | -0.098 | -0.138 |
|  | p value | 0.614 | 0.187 | 0.834 | 0.928 | 0.342 | 0.257 | 0.932 | 0.553 | 0.812 | 0.398 | 0.96 | 0.324 | 0.646 | 0.981 |  | 0 | 0.069 | 0.016 | 0.355 | 0.191 |
| sCD20 | r | 0.058 | -0.009 | -0.11 | -0.18 | -0.044 | 0.281** | 0.011 | 0.158 | 0.091 | 0.234* | -0.016 | 0.008 | 0.175 | 0.13 | 0.463** | 1 | 0.085 | 0.098 | -0.088 | -0.087 |
|  | p value | 0.586 | 0.929 | 0.298 | 0.086 | 0.677 | 0.007 | 0.919 | 0.134 | 0.389 | 0.025 | 0.878 | 0.938 | 0.095 | 0.218 | 0 |  | 0.421 | 0.355 | 0.405 | 0.41 |
| tCD3 | r | 0.214* | 0.241* | -0.178 | 0.164 | -0.025 | 0.141 | -0.125 | -0.157 | 0.012 | -0.183 | 0.137 | 0.19 | -0.283** | -0.316** | 0.19 | 0.085 | 1 | 0.826** | -0.514** | -0.665** |
|  | p value | 0.04 | 0.02 | 0.09 | 0.118 | 0.812 | 0.181 | 0.237 | 0.134 | 0.907 | 0.081 | 0.192 | 0.07 | 0.006 | 0.002 | 0.069 | 0.421 |  | 0 | 0 | 0 |
| sCD3 | r | 0.214* | 0.243* | -0.142 | 0.222* | 0.013 | 0.063 | -0.102 | -0.176 | -0.033 | -0.163 | 0.196 | 0.277** | -0.181 | -0.247* | 0.251* | 0.098 | 0.826** | 1 | -0.464** | -0.631** |
|  | p value | 0.04 | 0.019 | 0.177 | 0.034 | 0.905 | 0.553 | 0.332 | 0.094 | 0.751 | 0.12 | 0.061 | 0.008 | 0.084 | 0.017 | 0.016 | 0.355 | 0 |  | 0 | 0 |
| tFoxp3 | r | -0.106 | -0.231* | 0.085 | -0.153 | 0.203 | -0.186 | 0.092 | 0.116 | -0.022 | 0.246* | -0.278** | -0.238* | 0.202 | 0.182 | -0.098 | -0.088 | -0.514** | -0.464** | 1 | 0.618** |
|  | p value | 0.316 | 0.027 | 0.419 | 0.145 | 0.052 | 0.076 | 0.384 | 0.27 | 0.836 | 0.018 | 0.007 | 0.022 | 0.053 | 0.083 | 0.355 | 0.405 | 0 | 0 |  | 0 |
| sFoxp3 | r | 0.019 | -0.364** | 0.005 | -0.133 | 0.08 | -0.259* | 0.107 | 0.210* | -0.039 | 0.252* | -0.151 | -0.162 | 0.443** | 0.366** | -0.138 | -0.087 | -0.665** | -0.631** | 0.618** | 1 |
|  | p value | 0.856 | 0 | 0.96 | 0.206 | 0.447 | 0.013 | 0.312 | 0.045 | 0.711 | 0.015 | 0.15 | 0.123 | 0 | 0 | 0.191 | 0.41 | 0 | 0 | 0 |  |

*indicates *P* < 0.05, **indicates *P* < 0.001. The lowercase letters ‘t’ and ‘s’ before each marker represent expression levels or densities of this marker (or marker-positive TILs) in the tumor and stromal compartment, respectively. Abbreviations: Gal9, Galectin-9, TILs, tumor-infiltrating lymphocytes.

**Supplementary Table 7. Association between markers expression or their co-expression and LRFS of spinal chordoma patients**

| Factors | Categories | Numbers | Univariate analysis | |
| --- | --- | --- | --- | --- |
|  |  |  | χ^2^ | *P*-value |
| Tumoral HHLA2 expression | positive | 74 | 15.881 | **<0.001** |
|  | negative | 18 |  |  |
| Tumoral B7-H3 expression | positive | 10 | 1.834 | 0.176 |
|  | negative | 82 |  |  |
| Tumoral IDO-1 expression | positive | 19 | 0.355 | 0.551 |
|  | negative | 73 |  |  |
| Tumoral PD-L1 expression | positive | 79 | 2.218 | 0.136 |
|  | negative | 13 |  |  |
| Tumoral Galectin-9 expression | positive | 10 | 8.59 | 0.062 |
|  | negative | 82 |  |  |
| Stromal HHLA2 expression | positive | 76 | 1.655 | 0.198 |
|  | negative | 16 |  |  |
| Stromal B7-H3 expression | positive | 58 | 8.334 | 0.078 |
|  | negative | 34 |  |  |
| Stromal IDO-1 expression | positive | 74 | 4.217 | 0.428 |
|  | negative | 18 |  |  |
| Stromal PD-L1 expression | positive | 73 | 23.199 | **<0.001** |
|  | negative | 19 |  |  |
| Stromal Galectin-9 expression | positive | 66 | 13.446 | **<0.001** |
|  | negative | 26 |  |  |
| Tumoral HHLA2 and tPDL1 co-expression | positive | 70 | 5.628 | **0.018** |
|  | negative | 22 |  |  |
| Stromal B7H3 and Galectin-9 co-expression | positive | 60 | 0.598 | 0.439 |
|  | negative | 32 |  |  |
| Stromal B7H3 and IDO-1 co-expression | positive | 56 | 5.288 | **0.021** |
|  | negative | 36 |  |  |
| Stromal Galectin-9 and IDO-1 co-expression | positive | 61 | 1.059 | 0.303 |
|  | negative | 31 |  |  |

Bold values indicate *P* < 0.05; Abbreviations: LRFS, local recurrence-free survival; P value from the log-rank test was corrected as previously suggested.

**Supplementary Table 8. Association between markers expression or their co-expression and OS of spinal chordoma patients**

| Factors | Categories | Numbers | Univariate analysis | |
| --- | --- | --- | --- | --- |
|  |  |  | χ^2^ | *P*-value |
| Tumoral HHLA2 expression | positive | 74 | 32.979 | **<0.001** |
|  | negative | 18 |  |  |
| Tumoral B7-H3 expression | positive | 10 | 2.429 | 0.119 |
|  | negative | 82 |  |  |
| Tumoral IDO-1 expression | positive | 19 | 1.82 | 0.177 |
|  | negative | 73 |  |  |
| Tumoral PD-L1 expression | positive | 79 | 2.896 | 0.089 |
|  | negative | 13 |  |  |
| Tumoral Galectin-9 expression | positive | 10 | 2.963 | 0.664 |
|  | negative | 82 |  |  |
| Stromal HHLA2 expression | positive | 76 | 3.246 | 0.608 |
|  | negative | 16 |  |  |
| Stromal B7-H3 expression | positive | 58 | 23.337 | **<0.001** |
|  | negative | 34 |  |  |
| Stromal IDO-1 expression | positive | 74 | 3.647 | 0.527 |
|  | negative | 18 |  |  |
| Stromal PD-L1 expression | positive | 73 | 27.113 | **<0.001** |
|  | negative | 19 |  |  |
| Stromal Galectin-9 expression | positive | 66 | 34.494 | **<0.001** |
|  | negative | 26 |  |  |
| Tumoral HHLA2 and tPDL1 co-expression | positive | 70 | 4.249 | **0.039** |
|  | negative | 22 |  |  |
| Stromal B7H3 and Galectin-9 co-expression | positive | 60 | 3.665 | 0.056 |
|  | negative | 32 |  |  |
| Stromal B7H3 and IDO-1 co-expression | positive | 56 | 0.425 | 0.514 |
|  | negative | 36 |  |  |
| Stromal Galectin-9 and IDO-1 co-expression | positive | 61 | 3.63 | 0.057 |
|  | negative | 31 |  |  |

Bold values indicate *P* < 0.05; Abbreviations: OS, overall survival; P value from the log-rank test was corrected as previously suggested.
